# TNFR1 signaling converging on FGF14 controls neuronal hyperactivity and sickness behavior in experimental cerebral malaria

**DOI:** 10.1186/s12974-023-02992-7

**Published:** 2023-12-19

**Authors:** Nolan M. Dvorak, Nadia D. Domingo, Cynthia M. Tapia, Paul A. Wadsworth, Mate Marosi, Yosef Avchalumov, Chanida Fongsaran, Leandra Koff, Jessica Di Re, Catherine M. Sampson, Timothy J. Baumgartner, Pingyuan Wang, Paula P. Villarreal, Olivia D. Solomon, Sonja J. Stutz, Aditi, Jacob Porter, Komi Gbedande, Brendan Prideaux, Thomas A. Green, Erin H. Seeley, Parimal Samir, Kelley T. Dineley, Gracie Vargas, Jia Zhou, Irma Cisneros, Robin Stephens, Fernanda Laezza

**Affiliations:** 1 Department of Pharmacology & Toxicology, University of Texas Medical Branch, Galveston, TX 77555 USA; 2 Department of Internal Medicine, Division of Infectious Diseases, University of Texas Medical Branch, Galveston, TX 77555 USA; 3 Department of Pathology, University of Texas Medical Branch, Galveston, TX 77555 USA; 4 Department of Neurobiology, University of Texas Medical Branch, Galveston, TX 77555 USA; 5 Clinical Sciences Program, The Institute for Translational Sciences, University of Texas Medical Branch, Galveston, TX 77555 USA; 6 Center for Addiction Sciences and Therapeutics, University of Texas Medical Branch, Galveston, TX 77555 USA; 7 Department of Microbiology & Immunology, University of Texas Medical Branch, Galveston, TX 77555 USA; 8 Center for Immunity and Inflammation and Department of Pharmacology, Physiology and Neuroscience, Rutgers New Jersey Medical School, Newark, NJ 07301 USA; 9 Department of Chemistry, University of Texas, Austin, TX 78712 USA

**Keywords:** Ion channels, Neuroinflammation, Neuronal excitability, Phosphorylation, Synaptic plasticity, Therapeutic target, Hippocampus

## Abstract

**Background:**

Excess tumor necrosis factor (TNF) is implicated in the pathogenesis of hyperinflammatory experimental cerebral malaria (eCM), including gliosis, increased levels of fibrin(ogen) in the brain, behavioral changes, and mortality. However, the role of TNF in eCM within the brain parenchyma, particularly directly on neurons, remains underdefined. Here, we investigate electrophysiological consequences of eCM on neuronal excitability and cell signaling mechanisms that contribute to observed phenotypes.

**Methods:**

The split-luciferase complementation assay (LCA) was used to investigate cell signaling mechanisms downstream of tumor necrosis factor receptor 1 (TNFR1) that could contribute to changes in neuronal excitability in eCM. Whole-cell patch-clamp electrophysiology was performed in brain slices from eCM mice to elucidate consequences of infection on CA1 pyramidal neuron excitability and cell signaling mechanisms that contribute to observed phenotypes. Involvement of identified signaling molecules in mediating behavioral changes and sickness behavior observed in eCM were investigated in vivo using genetic silencing.

**Results:**

Exploring signaling mechanisms that underlie TNF-induced effects on neuronal excitability, we found that the complex assembly of fibroblast growth factor 14 (FGF14) and the voltage-gated Na^+^ (Na_v_) channel 1.6 (Na_v_1.6) is increased upon tumor necrosis factor receptor 1 (TNFR1) stimulation via Janus Kinase 2 (JAK2). On account of the dependency of hyperinflammatory experimental cerebral malaria (eCM) on TNF, we performed patch-clamp studies in slices from eCM mice and showed that *Plasmodium chabaudi* infection augments Na_v_1.6 channel conductance of CA1 pyramidal neurons through the TNFR1–JAK2–FGF14–Na_v_1.6 signaling network, which leads to hyperexcitability. Hyperexcitability of CA1 pyramidal neurons caused by infection was mitigated via an anti-TNF antibody and genetic silencing of FGF14 in CA1. Furthermore, knockdown of FGF14 in CA1 reduced sickness behavior caused by infection.

**Conclusions:**

FGF14 may represent a therapeutic target for mitigating consequences of TNF-mediated neuroinflammation.

**Supplementary Information:**

The online version contains supplementary material available at 10.1186/s12974-023-02992-7.

## Introduction

Neuroinflammatory syndromes arise from a complex interplay between the immune response, including the pro-inflammatory cytokine tumor necrosis factor (TNF), and neurons [1, 2]. In the brain, TNF can exert opposing functions, with elevated levels either stimulating or suppressing neuronal network activity [3] depending on disease context, brain regions, and signal transduction mechanisms [4–7]. There is compelling evidence supporting the direct influence of TNF on synaptic transmission [8–10] and intrinsic excitability [11–13] through the regulation of ion channels of neurons, leading to changes in brain network activity and behavioral outcomes [11, 14, 15]. Yet, the molecular signaling mechanisms underlying TNF-mediated changes in neuronal function remain underdefined.

In cerebral malaria (CM), infection of erythrocytes with *Plasmodium spp.* leads to elevated levels of TNF family members in the bloodstream, exposing the brain to an inflammatory milieu [16]. CM is characterized by reduced consciousness and is associated with seizures, which significantly increase the risk of mortality and predispose patients to epilepsy and other long-term neurological sequelae [17]. The severity of CM in humans has been linked to mutations in the promoter region of the TNF gene, as well as the ratio of TNF to the regulatory cytokine IL-10 [18]. However, clinical trials investigating the use of anti-TNF agents in humans have not been successful in preventing mortality and neurological complications [19], highlighting the need for mechanistic studies on the effect of *Plasmodium* infection on the brain and the signaling mechanisms underlying TNF-mediated neuronal regulation. *P. chabaudi* infection of IL-10-deficient mice is a highly validated TNF-dependent model of neuroinflammation [20–23]. This model recapitulates many of the features of *P. falciparum-*induced human cerebral malaria, including neuropathology with gliosis and severe intravascular congestion. In addition, sickness behaviors from this infection are inhibited by TNF neutralization [20, 23].

The voltage-gated Na + (Na_v_) channel isoform 1.6 (Na_v_1.6) is crucial for neuronal excitability and synaptic transmission in the hippocampus [24–26]. The α subunit of Na_v_1.6 enables ion conduction and action potential propagation, but its full physiological function relies on the interaction with the accessory protein fibroblast growth factor 14 (FGF14) [27–31]. In neurons, FGF14 serves as an intracellular signaling hub, which is dynamically regulated by kinases known to be effectors of TNF receptor signaling [32, 33]. For example, phosphorylation of FGF14^Y158^ by Janus Kinase 2 increases FGF14/Na_v_1.6 complex assembly, which leads to changes in the functional properties of the Na_v_1.6 channel that fine-tune Na^+^ conductance and neuronal firing [32]; however, upstream signal transduction mechanisms regulating JAK2’s phosphorylation of FGF14 are not well characterized. With a combination of a cell-based high-throughput screening, brain slice electrophysiology, and in vivo genetic silencing in an experimental cerebral malaria (eCM) model, we discovered a TNFR1–FGF14–Na_v_1.6 signaling network mediated by JAK2 and demonstrated its regulatory effects at the cellular, circuital, and behavior levels. Overall, these studies highlight a novel cytokine signaling response converging on FGF14 that contributes to neuronal and systemic effects of malaria, with mechanistic insight that is applicable to a broad array of neuroinflammatory conditions.

## Materials and methods

### Chemicals

D-luciferin was purchased from Gold Biotechnologies (St. Louis, MO, USA). TNF-α protein was purchased from Abcam (Cambridge, UK) and R-7050 was purchased from Selleck (Houston, TX, USA). Screened compounds are described below. Repurchased hits, including NVP-BSK805, momelotinib, and fedratinib, were obtained from Selleck. ZL181 used in this study was synthesized as previously described [29]. All salts for electrophysiological recordings were purchased from Sigma-Aldrich (St. Louis, MO, USA) unless otherwise noted.

### DNA constructs

The CLuc-FGF14^WT^, CLuc-FGF14^Y158A^, FGF14^WT^-NLuc, and CD4-Na_v_1.6 CTD^WT^-NLuc constructs used in LCA experiments were engineered and characterized as previously described [29, 31, 34–36].

### Cell culture

All cell culture reagents were purchased from Invitrogen (Carlsbad, CA, USA). HEK293 cells were maintained in a 1:1 mixture of Dulbecco’s Modified Eagle Medium and F-12 supplemented with 10% fetal bovine serum, 100 units/mL of penicillin, and 100 µg/mL streptomycin and incubated at 37 ℃ and 5% CO_2_. The HEK293 cell line double stably expressing CLuc-FGF14 and CD4-Na_v_1.6 CTD-NLuc was previously described [36] and maintained in the presence of additional antibiotics (0.5 mg/mL G418 and 5 µg/mL puromycin) to ensure stable expression. For transient transfection, cells were transfected at 80–90% confluence with equal amount (1 µg each) of plasmid pairs using Lipofectamine 3000 (Invitrogen, Waltham, MA, USA) according to the manufacturer’s instructions.

### The split-luciferase complementation assay (LCA)—96-well plate assay

Cells were trypsinized (0.25%), triturated in medium, and seeded in white, clear-bottom CELLSTAR μClear^®^ 96-well tissue culture plates (Greiner Bio-One, Monroe, NC, USA) at ~ 0.9 × 10^5^ cells per well in 200 μL of medium. For transiently transfected cells, the trypsinization occurred 48 h post-transfection. The cells were incubated for 24 h, and the growth medium was subsequently replaced with 100 μL of serum-free, phenol red–free DMEM/F12 medium (Invitrogen) containing inhibitors (0.25–50 μM). The final concentration of DMSO was maintained at 0.3% for all wells. Following 2 h incubation at 37 °C, the reporter reaction was initiated by injection of 100 μL substrate solution containing 1.5 mg/mL of D-luciferin dissolved in PBS (final concentration = 0.75 mg/mL) by the Synergy^™^ H4 Multi-Mode Microplate Reader (BioTek, Winooski, VT, USA). Luminescence readings were performed at 2-min intervals for 20 min, integration time 0.5 s, and the cells were maintained at 37 °C throughout the measurements. Signal intensity for each well was calculated as a mean value of peak luminescence; the calculated values were expressed as percentage of mean signal intensity of the per plate control samples.

### LCA—384-well plate assay

Cells were trypsinized (0.25%), triturated in a medium, and seeded in white, clear-bottom CELLSTAR μClear^®^ 384-well tissue culture plates (Greiner Bio-One) at 3 × 10^4^ cells per well in 40 μL of serum-free, phenol red–free DMEM/F12 medium using the Multidrop Combi (Thermo Fisher, Waltham, MA, USA). The LabCyte Echo 550 (SelectScience, Bath, UK) was used to acoustically deliver nanoliter volumes of compounds, TNF, and DMSO. For pathway screening, the Broad Collection was used, including 320 FDA-approved agents, clinical candidates, and small-molecule probes from the Informer Set that were commercially available. This library has been previously described as the “Informer Set” [37], which targets nearly 250 distinct proteins, encompassing a broad range of cell signaling pathways relevant to cancer cell line growth and survival. All compounds were provided in solution at 10 mM in DMSO and were screened at a final concentration of 30 μM (*n* = 1 compound per well; 320 compounds/plate), with the final concentration of DMSO maintained at 0.3% for all wells, as described previously [32]. To compare the effect of kinase inhibitors with and without the presence of simultaneous TNF stimulation, cells were incubated with either 1 ng/mL BSA or 1 ng/mL TNF for 1 h prior to addition of kinase inhibitors. Following a subsequent 2 h incubation at 37 °C, the reporter reaction was initiated by injection of 40 μL substrate solution containing 1.5 mg/mL of D-luciferin (final concentration = 0.75 mg/mL) by the Multidrop Combi (Thermo Fisher). After 1 h incubation, the Infinite M1000 (Tecan, Mannedorf, Switzerland) was used to detect luminescence.

### LCA data analysis

LCA data analysis was performed using the following equations:

1 $${{\text{Z}}}^{\mathrm{^{\prime}}}{\text{factor}}=1-3\mathrm{ x }\frac{{(\delta }_{p}+ {\delta }_{n})}{\left({\mu }_{p}- {\mu }_{n}\right)}$$

2 $$\mathrm{S }:{\text{B}}= \frac{{\mu }_{p}}{{\mu }_{n}}$$

3 $$\mathrm{S }:{\text{N}}= \frac{({\mu }_{\begin{array}{c}p \\ \end{array}}- {\mu }_{n})}{\sqrt{{\delta }_{p}^{2}}+ {\delta }_{n}^{2}}$$

4 $${\text{SW}}= \frac{{\mu }_{p}- {\mu }_{n}-3 x ({\delta }_{p}+ {\delta }_{n}) }{{\delta }_{p}}$$

where δ_*p*_ and δ_*n*_ are standard deviation of the positive control group *p* and the negative control group *n*, and μ_*p*_ and μ_*n*_ are the arithmetic means of the two groups, respectively; S:B, signal to background; S:N, signal to noise; and SW, signal window.

Z-scores were calculated for each screened compound using the following formula:

5 $${\text{Z}}-{\text{score}}= \frac{{\mu }_{i} - {\mu }_{DMSO} }{{\delta }_{DMSO}}$$

where μ_*i*_ is the luminescent signal of the sample (i.e., any particular screened compound), and μ_*DMSO*_ and δ_*DMSO*_ are the mean and standard deviation, respectively, of the per plate 0.3% DMSO controls for that sample. Each compound replicate was on an independent 384-well plate, and a percent luminescence and Z-score was calculated separately using each replicate's respective per plate controls. The two replicate percent luminescence values and Z-scores for each compound were subsequently averaged.

For hit dose–response validation studies, compounds were tested between 0.25 and 50 μM using *n* = 8 wells per concentration over two 384-well plates per compound. Luminescence was normalized to per plate 0.3% DMSO controls, and dose–response curves were obtained using GraphPad Prism 8 by fitting the data with a non-linear regression:

6 $${\text{A}}+ \frac{{\text{B}}-{\text{A}}}{1+{10}^{{\text{log}}\left({{\text{x}}}_{o}-{\text{x}}\right)}{\text{H}}}$$

where x is log10 of the compound concentration in M, x_*0*_ is the inflection point (IC_50_), A is the bottom plateau effect, B is the top plateau effect, and H is the Hill slope.

### Animals

B6.129P2-Il10^tm1Cgn^/J mutant (IL-10 KO, Jackson Laboratory (Bar Harbor, ME, USA)) male mice between the ages of 6 and 18 weeks were used for all experiments except where noted for behavioral studies, which included uninfected wild-type C57BL/6J mice as an internal control. Animals were also bred in The University of Texas Medical Branch Animal Resource Center. Mice were housed, *n* ≤ 5 per cage, with food and water ad libitum. Mice were closely monitored for health and overall well-being daily by veterinary staff and the investigators. Animal care and experiments were performed in accordance with US National Institutes of Health (NIH) guidelines and were approved by the Institutional Animal Care and Use Committee.

### Parasite and infection

Frozen stocks of *Plasmodium chabaudi chabaudi* (AS)-infected RBCs (iRBC) generously provided by Jean Langhorne and Patrick Duffy (NIH) were stored in liquid nitrogen [20]. Parasites were thawed and passed into C57BL/6 J (WT) mice. Parasitized blood from pass animals was diluted into an infection stock in Krebs–Ringer bicarbonate buffer (Sigma-Aldrich) on ice, followed by final dilution in normal saline to deliver 1 × 10^5^ iRBCs in 200 μl into experimental mice via intraperitoneal (IP) injection. Thin blood smears were collected on the morning of day 7 post-infection to monitor peripheral parasitemia. Slides were stained with Giemsa stain (Ricca Chemical Company, Arlington, TX, USA) diluted in PBS and counted on a light microscope (Nikon eclipse 80i). Animals with parasitemia exceeding 10% on day 7 p.i. were used in the study.

### RNA and protein extraction

Brains were rapidly extracted, and right and left hippocampi were dissected and pooled together in the same microcentrifuge tube containing RNALater (Invitrogen, AM7020) and kept in a −80 °C freezer until used. Tissue was homogenized using a TissueLyser II (Qiagen, Germantown, MD, USA) for 30 s at a speed of 30 Hz in 200–500 µL of ice-cold homogenization/extraction buffer (20 mM HEPES, 200 mM NaCl, 1 mM EDTA, 1 mM DTT, 10 µL/mL phosphatase inhibitor cocktail 2 (Sigma, Cat #P5726), 10 µL/mL phosphatase inhibitor cocktail 3 (Sigma, Cat #P0044), RNase inhibitor). Homogenized samples were aliquoted for RNA and protein isolation. The homogenized samples were centrifuged at 10,000 × g for 5 min at 4 °C and the supernatant was transferred to a new tube. The total RNA was prepared using a standardized protocol based on TRIzol reagent (Invitrogen). The amount of RNA was assessed by using Nanodrop equipment (DeNovix DS-11 + Spectrophotometer). The protein was lyzed in Tissue extraction reagent (Invitrogen, FNN0071) in a volume of 100 µl. The protein concentration was measured using a Precision Red, Advance Protein Assay Reagent #2 (Cytoskeleton, Inc. Denver, CO, USA) following the manufacturer’s instructions.

### Serum preparation

Blood was collected in BD Microtainer tubes (Becton Dickinson, Franklin Lakes, NJ, USA). After the collection of the whole blood, the blood was allowed to clot at room temperature for 30 min. The clotted blood was then centrifuged for 10 min at 2,000 × g. Serum was then collected and stored at −20 °C.

### Western blot analysis

For each sample, an equal amount of protein (30 µg) was loaded onto 4–15% SDS polyacrylamide gels and blotted onto a PVDF membrane (Bio-Rad, Hercules, CA, USA) using a Bio-Rad Mini-Blot transfer apparatus. Immunoblotting was performed at 4 °C overnight using primary antibodies directed TNF-α (D2D4) XP^®^ (dilution 1:500, Cell signaling, 11948) and Beta-actin (13E5) (dilution 1:3,000, Cell signaling, 5125S). Membranes were then incubated with a 1:1,500 of secondary antibody (Goat anti-rabbit IgG HRP, 7074P2, Cell signaling Technology) or 1:1,500 of horse anti-mouse IgG HRP (7076S, Cell signaling Technology) at room temperature for 1 h. Membranes were visualized using a chemiluminescence assay with Clarity Western ECL substrate (Bio-Rad) and imaged on a BioRad ChemiDoc™ MP Imaging system (Bio-Rad).

### Enzyme-linked immunosorbent assay (ELISA)

ELISA mouse TNF-α (DY410), and mouse IFN-γ (DY485) were performed according to the manufacturer's instructions (DuoSet ELISA development systems, R&D systems, Inc., Minneapolis, MN, USA). Briefly, a 96-well plate was coated overnight with mouse TNF-α or mouse IFN-γ capture antibody and then sequentially incubated with 1% BSA in PBS (pH 7.2) for 1 h, equal concentrations of protein lysates (150 µg) and standards, or 1:2 diluted samples of serum for 1 h, and streptavidin-HRP for 1 h. Plates were developed with TMB substrate and the enzymatic reaction was quantified by measuring absorbance at 450 nm using a GloMax Discover Microplate reader (Promega, Madison, WI, USA).

### Acute brain slice preparation following *P. chabaudi* infection

Electrophysiological recordings were performed in acutely prepared coronal brain slices containing the hippocampus 7 days after infection with *P. chabaudi*. For brain slice preparation, mice were anesthetized using isoflurane (Baxter, Deerfield, IL, USA) and quickly decapitated. After decapitation, brains were dissected and 300 µM coronal slices containing the hippocampus were prepared with a vibratome (Leica Biosystems, Buffalo Grove, IL, USA) in a continuously oxygenated (mixture of 95% O_2_/5% CO_2_) and chilled Tris-based artificial cerebrospinal fluid (aCSF) containing the following salts: 72 mM Tris–HCl; 18 mM Tris-Base; 1.2 mM NaH_2_PO_4_, 2.5 mM KCl; 20 mM HEPES; 20 mM sucrose; 25 mM NaHCO_3_; 25 mM glucose; 10 mM MgSO_4_, 3 mM Na pyruvate; 5 mM Na ascorbate; and 0.5 mM CaCl_2_ (pH = 7.4 and osmolarity = 300–310 mOsm). Prepared slices were first transferred to a continuously oxygenated and 31ºC recovery chamber containing fresh Tris-based aCSF for 15 min. After 15 min, slices were transferred to a continuously oxygenated and 31℃ chamber containing standard aCSF, which comprised the following salts: 123.9 mM NaCl; 3.1 mM KCl; 10 mM glucose; 1 mM MgCl_2_; 2 mM CaCl_2_; 24 mM NaHCO_3_; and 1.16 mM NaH_2_PO4 (pH = 7.4 and osmolarity = 300–310 mOsm). Slices were allowed at least 30 min of recovery prior to the start of recording.

### Patch-clamp electrophysiology—general

Borosilicate glass pipettes (Harvard Apparatus, Holliston, MA, USA) with resistance of 3 – 5 MΩ were fabricated using a PC-100 vertical Micropipette Puller (Narishige International Inc., East Meadow, NY, USA). Recordings were obtained using an Axopatch 200B amplifier (Molecular Devices, Sunnyvale, CA, USA). Membrane capacitance and series resistance were estimated using the dial settings on the amplifier, and capacitive transients and series resistance were compensated by 70–80%. Data acquisition and filtering occurred at 20 and 5 kHz, respectively, before digitization and storage. Clampex 9 software (Molecular Devices) was used to set experimental parameters, and electrophysiological equipment was interfaced to this software using a Digidata 1320A analog–digital interface (Molecular Devices). Analysis of electrophysiological data was performed using Clampfit 11 software (Molecular Devices) and GraphPad Prism 8 software (La Jolla, CA, USA). Results were expressed as individual replicates with SEM error bars. Statistical significance was determined using a one-way ANOVA with post hoc Tukey’s multiple comparisons test, with *p* < 0.05 being considered statistically significant.

### Whole-cell voltage-clamp recordings

Whole-cell voltage-clamp recordings to assess the Na_v_1.6-mediated *I*_Na_ of CA1 pyramidal cells in the slice preparation were performed as previously described [38]. Briefly, slices were transferred to a recording chamber perfused with continuously oxygenated and heated standard aCSF that was supplemented with 120 µM CdCl_2_, 1 µM ICA12142, and 10 nM Phrixotoxin3 to block currents mediated by voltage-gated Ca^2+^ channels, Na_v_1.1 channels, and Na_v_1.2 channels, respectively. Voltage-clamp recordings of CA1 pyramidal cells were performed using an internal solution comprising the following salts: 100 mM Cs-gluconate (Hello Bio, Princeton, NJ, USA); 10 mM tetraethylammonium chloride; 5 mM 4-aminopyridine; 10 mM EGTA; 1 mM CaCl_2_; 10 mM HEPES; 4 mM Mg-ATP; 0.3 mM Na_3_-GTP; 4 mM Na_2_-phosphocreatine, and 4 mM NaCl (pH = 7.4 and osmolarity = 285 ± 5 mOsm/L; CsOH was used to adjust pH and osmolarity). After GΩ formation and entry into the whole-cell configuration, the following cocktail of synaptic blockers was perfused for 1 min to mitigate synaptic currents: 20 µM bicuculline, 20 µM NBQX, and 100 µM AP-5 (synaptic blockers were purchased from Tocris, Bristol, UK). *I*_Na_ was elicited using the voltage-clamp protocols described elsewhere [39, 40] and data analysis was performed as previously described [38, 41].

### Whole-cell current-clamp recordings

Whole-cell current-clamp recordings to assess intrinsic firing properties of CA1 pyramidal cells were performed as previously described [38]. Briefly, slices were transferred to a recording chamber perfused with continuously oxygenated and heated standard aCSF. Current-clamp recordings of CA1 pyramidal cells were performed using an internal solution that comprised the following salts: 145 mM K-gluconate; 2 mM MgCl_2_; 0.1 mM EGTA; 2.5 mM Na_2_ATP; 0.25 mM Na_2_GTP; 5 mM Na_2_-phosphocreatine; and 10 mM HEPES (pH = 7.2 and osmolarity = 290 mOsm). After GΩ formation and entry into the whole-cell configuration, the amplifier was switched to *I* = 0 mode for approximately 1 min to determine the resting membrane potential before switching to current-clamp mode to assess intrinsic excitability. During this 1-min interval in *I* = 0 mode, the same cocktail of synaptic blockers used during voltage-clamp recordings was perfused to mitigate synaptic currents. To assess intrinsic excitability, evoked responses were measured in response to a range of current injections from -20 pA to + 180 pA. Current steps were 800 ms in duration, and the change in the injected current between steps was 10 pA. Analysis of current-clamp recordings was then performed as previously described [42].

### Anti-TNF antibody treatment

Mice were injected IP with 0.2 µg of monoclonal anti-mouse TNF antibody (clone XT3.11, catalog # BE0058; BioXCell, Lebanon, NH, USA) or IgG1 isotype control, anti-horseradish peroxidase antibody (catalog # BE0088) in 200 µL sterile saline daily from day 5 p.i. to day 7 p.i. This clone inhibits TNF and the highly related cytokine lymphotoxin.

### Construction of viral vector knocking down FGF14

Five hairpins were designed using the strategy of Crofton et al. [43] and oligonucleotides inserted into AAV plasmids expressing GFP from a CMV promoter and the hairpin from a mouse U6 pol3 promoter. The 24 base stems were connected with a 10 base miRNA loop (CCTTCCTGTCA). After sequence verification, each of the 5 shRNA constructs were tested for knockdown efficiency in HEK293 cells co-transfected with a plasmid overexpressing mouse FGF14 at a ratio of 9:1 (shRNA:overexpressing). Efficiency was determined by qPCR using primers targeted to mouse FGF14. The most effective shRNA was construct #4 (ATAGCAATGAATGGAGAAGGTTAC) at > 90% inhibition (Additional file 1: Figure S1), similar to the efficiency of our previous constructs [44–47]. The shFGF14 and shCTRL [46] plasmids were sent to the UNC Vector Core for packaging into AAV2 capsid. Viral titer was determined using dot blot analysis and ranged from 1 × 10^10.2^ to 1 × 10^12^. Functional knockdown was verified ex vivo by electrophysiological analysis (Additional file 1: Figure S2).

### In vivo genetic silencing of FGF14

In order to knockdown FGF14 in vivo, mice were anesthetized with isoflurane (Baxter) and injected bilaterally with control vector (AAV-shCTRL) or AAV-shFGF14 into the CA1 region of the hippocampus (1 µL/side over 10 min) using stereotaxic coordinates (AP = −2.3, L = 1.7, V =  − 1.5). After injection of 1 µL bilaterally, the needles remained in place for 10 addition min in order to allow for spread of the vector and to prevent the vector from spreading up the needle track as previously described [45]. Accurate placements were confirmed following brain extraction by visualizing the green fluorescent protein signal with a Dual Fluorescent Protein Flashlight and VG2 barrier filter glasses (Nightsea, Bedford, MA, USA) [48]. No animals were excluded based upon vector placement. Patch-clamp recordings were performed only from pyramidal neurons expressing GFP in the CA1 region.

### Fluorescence imaging

Widefield fluorescence microscope images were acquired with a Luigs & Neumann (Ratingen, Germany) microscope equipped with 470 nm LED illumination (Cool LED (Andover, Hampshire, UK) *p*E-4000), used to excite GFP. Excitation and emission were separated using a GFP filter cube (Chroma (Bellows Falls, VT, USA) 49002-Et-EGFP) with detection by a CCD camera (Hamamatsu (Shizuoka, Japan) Orca-Fusin BT). Individual overlapping images (2304 × 2304 pix, 16 bit) were taken with a 4 × 0.1 N.A. objective (Olympus (Tokyo, Japan) UIS2 4 × Plan N). The images were fused and transformed to RGB offline with Adobe Photoshop.

### Confocal imaging

Confocal images were acquired with a Zeiss LSM-880 (Jena, Germany) confocal microscope with a 20 × 0.8 N.A. objective. A multi-track acquisition was done with excitation lines at 405 nm for DAPI and 488 nm for green fluorescence protein (GFP). Respective emission filters were band-pass 410–479 nm, and band-pass 494–550 nm. Tiled Z-stack images were acquired to cover the entire thickness and region of interest (whole brain slice, single hemisphere, or hippocampus). All acquisition parameters, including photomultiplier gain and offset, were kept constant throughout each set of experiments. Acquired Z-stacks were sum-projected using Fiji ImageJ [49].

### Evoked extracellular field potential recordings

For extracellular field recordings, mice were anesthetized using isoflurane (Baxter), quickly decapitated, and then brains were quickly dissected in ice-cold aCSF containing: 125 mM NaCl, 25 mM glucose, 25 mM NaHCO_3_, 2.5 mM KCl, 2 mM CaCl_2_, 1.25 mM NaH_2_PO_4_, and 1 mM MgCl_2_. 400 µM coronal slices containing the hippocampus were prepared using a vibratome (Leica Biosystems). 2–3 slices were immediately transferred to a submerged-type recording chamber (Kerr Scientific Instruments, Christchurch, NZ) and incubated at room temperature for at least 2 h, with remaining slices being stored in a submerged-type bath (Brain Slice Keeper, BSK4, Scientific Systems Design Inc, Mississauga, Ontario, Canada) filled with aCSF until use. Slices were constantly perfused at a rate of 2 mL/min with oxygenated aCSF maintained at room temperature. Recordings were conducted in the CA1 region of the hippocampus after 2 h of incubation. Evoked field excitatory post-synaptic potentials (fEPSP) were recorded from the CA1 stratum radiatum region of the hippocampus using a single Teflon-coated 50 µm tungsten wire electrode and stimulation was delivered to the CA3 region using a bipolar Teflon-coated 50 µm tungsten wire electrode connected to a stimulus isolator (ISO-Flex, A.M.P.I, Jerusalem, Israel) at the baseline rate of 0.033 Hz. Stimulation intensity was set at 30–40% of saturating intensity. Input/output was used to determine maximum response and general slice health. For each slice, an input/output curve was constructed from 10–80 V, and paired-pulse facilitation was recorded at 50 ms delay. After stable baseline recordings of 20–30 min, high-frequency stimulation at 100 Hz for 1 s was used and delivered as a long-term potentiation (LTP)-induction protocol, and LTP was measured as the relative fEPSP slope post high-frequency stimulation compared to pre-high frequency stimulation. Slices were discarded if the baseline was not stable for at least 15 min prior to the LTP protocol. Data are represented as mean with SEM error bars, and one-way ANOVA with post hoc Tukey’s multiple comparisons test was used to determine significance, with *p* < 0.05 being considered statistically significant.

### Modified SHIRPA assessment

Beginning on day 5 p.i., daily assessments were performed on all animals using an abbreviated version of the modified SmithKline Beecham, Harwell Imperial College, Royal London Hospital Phenotype Assessment (SHIRPA) protocol [50]. The abbreviated SHIRPA involves nine semi-quantitative tests for general health, sensory function, baseline behaviors, and neurological function. The assessment was done in a viewing jar and open arena and took approximately 5 min per mouse. First, the animal was placed in the viewing jar on top of a grid for 3 min. The following behaviors were recorded without disturbing the animal: body position, from 0 (completely flat) to 5 (repeated vertical leaping); spontaneous activity, from 0 (none, resting) to 4 (extremely rapid/darting movement). From the viewing jar, the animal is transferred and dropped onto the arena floor. Then, additional behaviors were recorded: palpebral closure, which ranged from 0 (eyes closed) to 2 (eyes wide open); gait, ranging from 0 (incapacitated) to 3 (normal); tail elevation during forward motion, ranging from 0 (dragging) to 2 (elevated tail); touch escape, assessed with finger stroke and scores range from 0 (no response) to 3 (vigorous escape response to approach); qualitative grip strength, measured by lowering animal, allowing it to grip the grid and applying a gentle backward pull, with scores ranging from 0 (none) to 4 (unusually effective). Next, during supine restraint, the animal is firmly scruffed, and heart rate scored by palpation below the sternum, with scores ranging from 0 (slow, bradycardia) to 2 (fast, tachycardia). Lastly, righting reflex is measured by putting the mouse back into the open arena and observing the landing position, with scores ranging from 0 (rights itself) to 3 (no impairment). A healthy, uninfected IL-10 KO or WT mouse’s expected score ranges from 21 to 23, while mice are predicted to die when the score is less than or equal to 16 before day 9 p.i [23]. This method also converges with a behavioral battery developed mathematically to determine experimental cerebral malaria in *P. berghei* ANKA infected animals [51].

### Evaluation of body temperature and weight

Body temperature was assessed during the course of infection using rounded stainless steel rectal probes and a BIO-TK8851 digital rodent model thermometer (Bioseb, Pinellas Park, FL, USA). Between each use, probes were sanitized with CaviCide (Metrex Research Corp., Romulus, MI, USA). Animal mass measured using an OHAUS Scout Pro SP601 portable balance (OHAUS, Parsippany, NJ, USA).

## Results

### Increased assembly of the FGF14/Na_v_1.6 complex mediated by the TNFR1–JAK2 pathway

**Fig. 1:**
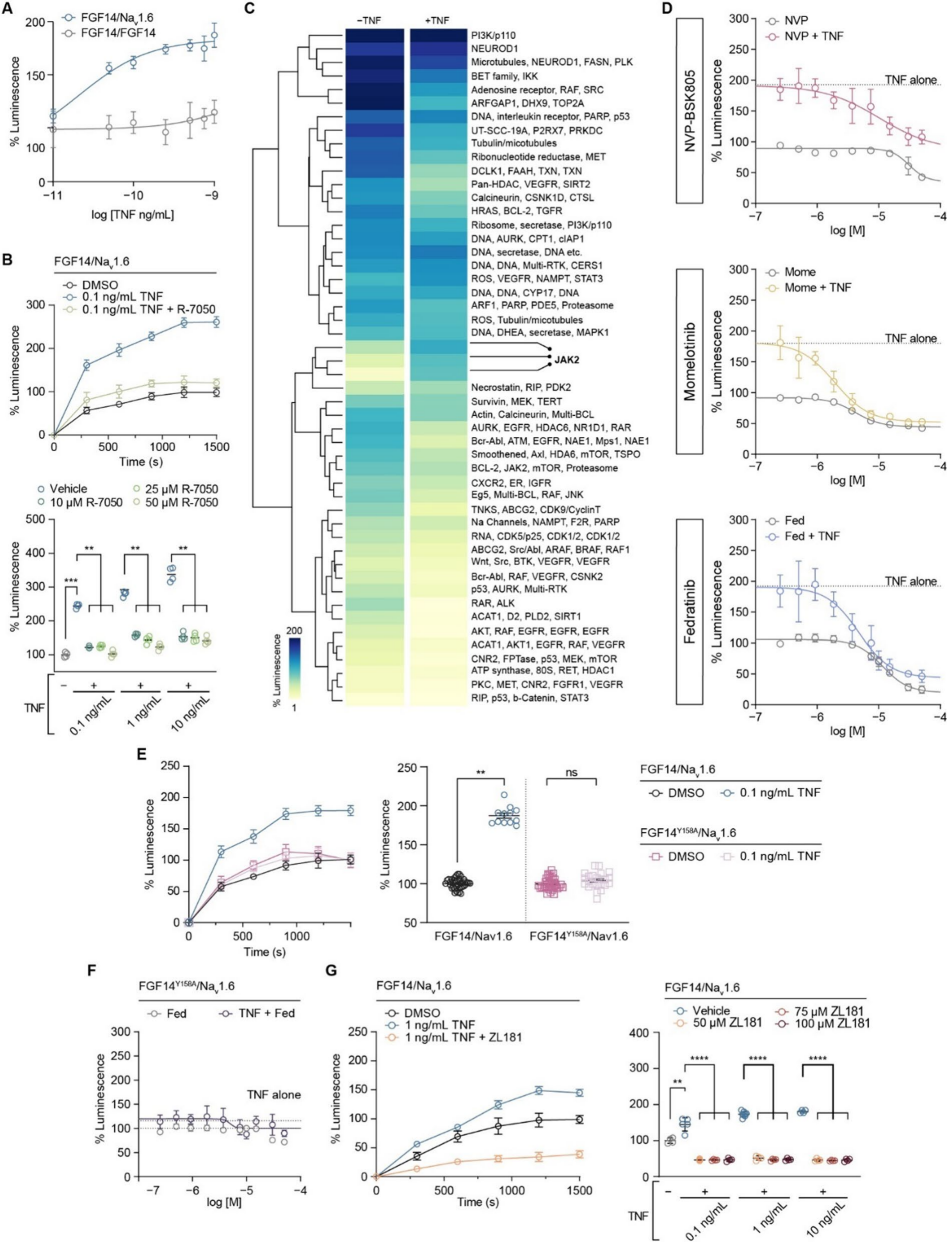
Increased assembly of the FGF14/Na_v_1.6 complex mediated by the TNFR1–JAK2 pathway. **A** Comparison of the effects of increasing concentrations of TNF on FGF14/Na_v_1.6 complex assembly (*n* = 10–12 biological replicates/group) and FGF14/FGF14 complex assembly (*n* = 20 biological replicates/group). **B** Top: percentage luminescence in the split-luciferase complementation assay (LCA) as a function of time for the indicated experimental groups. Bottom: comparison of the effects of TNF on FGF14:Na_v_1.6 complex assembly in the presence of increasing concentrations of TNF and the TNFR1 antagonist R-7050 (*n* = 4 biological replicates/group). **C** Heatmap summary of high-throughput screening of kinase inhibitors against FGF14/Na_v_1.6 complex assembly. **D** Dose–response analyses of three structurally diverse JAK2 inhibitors against FGF14/Na_v_1.6 complex assembly with and without TNF pre-treatment (*n* = 4–5 biological replicates/group). **E** Left: percentage luminescence in the LCA as a function of time for the indicated experimental groups. Right: effects of 1 ng/mL TNF treatment on FGF14/Na_v_1.6 and FGF14^Y158A^/Na_v_1.6 complex assembly (*n* = 12–38 biological replicates/group). **F** Dose–response analyses of the JAK2 inhibitor fedratinib on FGF14.^Y158A^/Na_v_1.6 complex assembly with and without TNF stimulation (*n* = 4–5 biological replicates/group). **G** Left: percentage luminescence in the LCA as a function of time for the indicated experimental groups. Right: effects of ZL181 treatment on the TNF-induced increase in FGF14:Na_v_1.6 complex assembly (*n* = 4–8 biological replicates/group). Data are represented as mean ± SEM. In (**B**, bottom and **G**, right) significance was assessed using a 2-way ANOVA with post hoc Dunnett’s multiple comparisons test. In (**E**, right), significance was assessed using a Student’s t-test (*ns* not significant; **p < 0.01; ***p < 0.001; ****p < 0.0001)

The full physiological function of the Na_v_1.6 channel relies on interactions between its α subunit and auxiliary proteins, including FGF14 [30, 31, 52]. The complex formation of FGF14 with the C-terminal domain (CTD) of the Na_v_1.6 channel is regulated by phosphorylation of FGF14 by kinases [32, 33] whose activities can be regulated by activation of cytokine receptors, including TNFR1 [53]. To investigate potential convergence between signaling pathways upstream of FGF14 and downstream of TNFR1, we treated HEK293 cells stably expressing CLuc-FGF14 and CD4-Na_v_1.6-CTD-NLuc constructs with soluble TNF protein. Treatment of these cells (hereafter referred to as Clone V cells) with TNF caused a dose-dependent increase in FGF14/Na_v_1.6 complex assembly detected through the split-luciferase complementation assay (LCA) (Fig. 1A). In contrast, TNF treatment had negligible effects on the assembly of the FGF14/FGF14 homodimer (Fig. 1A), a protein complex that forms in cells and is also regulated by phosphorylation [31, 32]. To test if the effect of TNF on FGF14/Na_v_1.6 complex assembly was mediated by TNFR1 activation, which is endogenously expressed in HEK293 cells [54], the experiments were replicated with varying concentrations of the TNFR1 antagonist R-7050. These studies found that R-7050 blocked TNF-induced enhancement of FGF14/Na_v_1.6 complex assembly, restoring it to the level observed in the untreated control condition (Fig. 1B).

In order to identify signaling molecules that could potentially converge to produce the observed phenotype, we used the LCA to conduct a differential high-throughput screening (HTS) of kinase inhibitors. The screening was conducted using Clone V cells treated with an inert protein (bovine serum albumin) or TNF, and cells were treated with vehicle (dimethyl sulfoxide; DMSO) or compounds from the Broad Institute Collection, which includes 320 compounds with known toxicity profiles, established mechanisms of action, and internal annotations of targets and cellular signaling pathways (Additional file 1: Figure S3A). The coefficient of variation (CV) and Z' factor for our in-cell HTS assay were found to be within an acceptable range (control (*n* = 4 plates): CV = 0.09 ± 0.01 and *Z*’ = 0.78 ± 0.06; TNF-stimulated (*n* = 2 plates): CV = 0.06 ± 0.002 and *Z*’ = 0.72 ± 0.01; data are mean ± SD). Hierarchical clustering of LCA signals revealed a distinct pattern of activity with hits targeting Janus kinase 2 (JAK2) preventing TNF-induced potentiation of FGF14/Na_v_1.6 assembly (Fig. 1C). To validate the results of the HTS, three structurally diverse JAK2 inhibitors were chosen for confirmatory dose-responses (Fig. 1D), and the JAK2-selective inhibitor fedratinib was selected for further mechanistic studies based on potency. Whereas JAK2 inhibitors blocked the effects of TNF on FGF14/Na_v_1.6 complex assembly and restored complex assembly to a level comparable to the unstimulated control condition, the IKK inhibitor CAY10576 did not occlude the increase in complex assembly induced by TNF (Additional file 1: Figure S3B), suggesting that regulation of the complex assembly by TNFR1 is selective for the TNFR1–JAK2 pathway.

Previously, we demonstrated that JAK2 regulates FGF14/Na_v_1.6 complex assembly by directly phosphorylating FGF14 at Y158 [32]. Based on this finding, we hypothesized that FGF14^Y158^ might function as a convergence point within the identified TNF signaling pathway. To this end, we conducted additional LCA experiments in cells transiently expressing the FGF14^Y158A^ phosphosilent mutant (CLuc-FGF14^Y158A^) along with the CD4-Na_v_1.6 CTD-NLuc construct, with TNF alone or in combination with Fedratinib. We found that TNF treatment had no effect on FGF14/Na_v_1.6 complex assembly when assayed in the presence of the FGF14^Y158A^ mutant (Fig. 1E). In addition, the effect of fedratinib on complex assembly in the presence or absence of TNF treatment was significantly blunted when assayed in the presence of the FGF14^Y158A^ mutant (Fig. 1F). Additionally, we showed that pharmacological occupancy of FGF14^Y158^ with ZL181, a previously validated inhibitor of FGF14 [29], blocks the effect of TNF on FGF14/Na_v_1.6 complex assembly (Fig. 1G), providing evidence that FGF14^Y158^ is the convergence point of the TNF-mediated signal transduction mechanism that regulates the Na_v_1.6 channel macromolecular complex assembly. Collectively, these findings reveal a novel TNF signaling mechanism where FGF14 is a target of TNFR1 stimulation through JAK2-mediated phosphorylation of FGF14, which increases the interaction of FGF14 with the Na_v_1.6 channel.

### Mechanisms of TNF-mediated neuronal dysfunction in hyperinflammatory eCM

FGF14 and Na_v_1.6 are both highly expressed in hippocampal pyramidal neurons, exhibiting strong immunoreactivity and co-localization at the axon initial segment, soma, and dendrites [35]. Genetic deletion, genetic mutation, and pharmacological inhibition of FGF14 lead to decreases in Na_v_ channel conductance and intrinsic firing of neurons [27, 30, 32, 55, 56], whereas overexpression of FGF14 produces contrasting effects [57], highlighting the importance of the FGF14/Na_v_1.6 interaction in fine-tuning neuronal output. Phosphorylation signaling cascades play a crucial role in regulating the function of the FGF14/Na_v_1.6 complex [32, 33, 35], and levels of Na_v_1.6 channel macromolecular complex phosphorylation vary as a result of changes in receptor signal transduction that can lead to changes in neuronal excitability [58]. Therefore, we investigated the relationship between the function of the FGF14/Na_v_1.6 complex and cytokine signaling, which drives neuroinflammation.

**Fig. 2:**
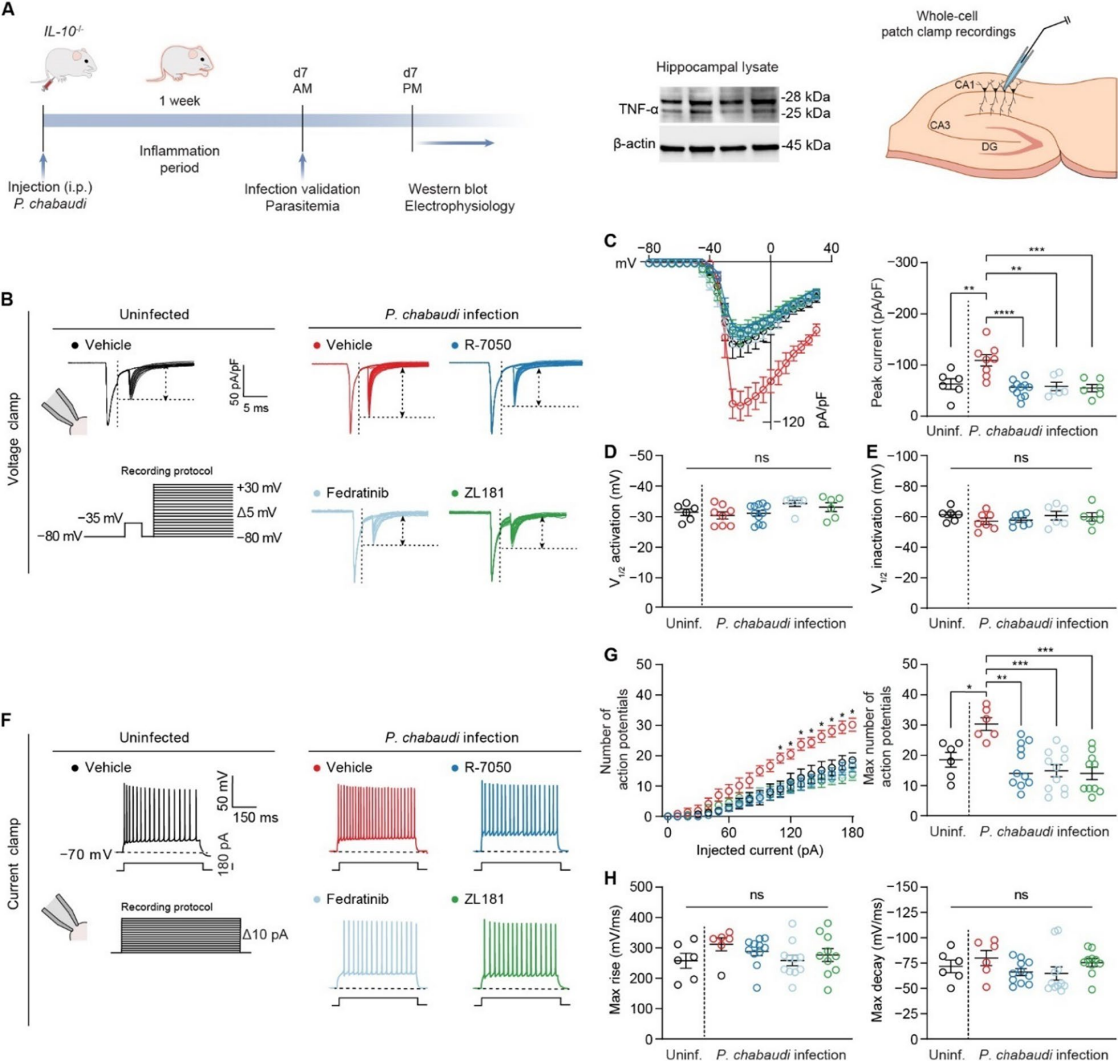
Mechanisms of TNF-mediated neuronal dysfunction in hyperinflammatory eCM. **A** Left: timeline for *P. chabaudi* infection and subsequent downstream analyses. Middle: presence of TNF protein in the hippocampus of *IL*-*10*^*−/−*^ mice infected with *P*. *chabaudi* on day 7 p.i. (lysate collected from *n* = 4 mice). Right: diagrammatic representation of whole-cell patch-clamp recording in CA1 pyramidal neurons. **B** Representative traces of Na_v_1.6-mediated *I*_Na_ elicited by CA1 pyramidal neurons in response to the depicted voltage-clamp protocol in slices from the indicated experimental groups. **C** Comparison of current–voltage relationships of CA1 pyramidal neurons among the indicated experimental groups. **D** Comparison of V_1/2_ of activation of Na_v_1.6-mediated *I*_Na_ among the indicated experimental groups. **E** Comparison of V_1/2_ of inactivation of Na_v_1.6-mediated *I*_Na_ among the indicated experimental groups. **F** Representative traces of action potentials fired by CA1 neurons at the 180-pA injected current step. **G** Comparison of action potential discharge among the indicated experimental groups. **H** Comparison of action potential kinetics among the indicated experimental groups. Data are mean ± SEM (*n* = 6–11 neurons/group; recordings were performed in slices from at least *N* = 3 mice per group). Significance was assessed using a one-way ANOVA with post hoc Tukey’s multiple comparisons test. In (**C**, right, **D**, **E**, **G**, right, and **H**): ns, not significant; *p < 0.05; **p < 0.01; ***p < 0.001; ****p < 0.0001. In (**G**, left), * denotes current steps at which the number of action potentials fired by CA1 pyramidal neurons in slices from infected mice treated with vehicle is significantly greater (*p* is at least < 0.05) than the number of action potentials fired by CA1 neurons in other experimental groups

Hyperinflammatory experimental cerebral malaria (eCM) is induced by infection of mice deficient in the regulatory cytokine IL-10 (*IL-10*^*−/−*^) with *Plasmodium chabaudi* [20]. While in wild-type animals *P. chabaudi* is a mild infection, in this genetic background, *P. chabaudi* infection leads to lethal neuroinflammation dependent on TNF signaling for mortality [20]. We used this model rather than infection of C57BL/6J wild-type mice with *P. berghei* ANKA to allow us to determine if there are inflammation-specific effects on neurons in eCM. *P. berghei* ANKA infection drives a more complex and severe form of eCM thought to be driven by virulence factors of the parasite strain itself, in addition to effects of the immune response elicited by the infection. Supporting the relevance and importance of the hyperinflammatory model, the balance of pro-inflammatory and regulatory cytokines, namely TNF and IL-10, and genetic variants in these genes, correlate with severity in human cerebral malaria [18, 59, 60]. Signs of hyperinflammation in the hippocampus can be neutralized by antibodies that block TNF, as illustrated by reduced glial activation, vascular coagulation, and behavioral changes associated with the disease [23]. Western blotting showed high levels of TNF in the hippocampus of eCM mice (Fig. 2A, Additional file 1: Figure S4), as seen systemically in this model [20]. Therefore, we hypothesized that infection of *IL*-*10*^*−/−*^ mice with *P*. *chabaudi* would lead to increased neuronal activity through the TNFR1–JAK2–FGF14–Na_v_1.6 signaling network.

To test this hypothesis, we performed whole-cell patch-clamp recordings in acute brain slices prepared from *IL-10*^*−/−*^ mice with hyperinflammatory eCM (Fig. 2A) on day 7 post-infection (p.i.). We first assessed the level of transient Na + current (*I*_Na_) of CA1 pyramidal neurons in slices from infected *IL-10 *^*−/−*^ mice to determine if this parameter was altered compared to neurons from uninfected *IL-10 *^*−/−*^ mice (Fig. 2B, Additional file 1: Table S1). To record *I*_Na_ of CA1 pyramidal neurons in slices, we used a previously described dual-step voltage-clamp protocol developed to mitigate space-clamp issues [40]. To isolate Na_v_1.6-mediated *I*_Na_, all recordings were performed in the presence of blockers of other Na_v_ channel isoforms present in CA1 pyramidal neurons. These voltage-clamp recordings revealed that *P*. *chabaudi* infection of *IL*-*10*^−/−^ mice significantly augmented the Na_v_1.6-mediated *I*_Na_ density of CA1 pyramidal neurons (Fig. 2C, Additional file 1: Table S1). Notably, neither *P. chabaudi* infection nor any inhibitors affected the voltage-dependence of activation (Fig. 2D) or inactivation (Fig. 2E) of these currents (Additional file 1: Table S1). This indicates that the influence of *P. chabaudi* infection is likely exerted primarily through alterations in the trafficking of channels to the cell surface, rather than by modifying their biophysical properties. Next, we performed the same recordings after treatment of slices with the TNFR1 antagonist R-7050 (Fig. 2B). Acute (ex vivo) treatment of slices from the eCM mice with R-7050 restored the Na_v_1.6-mediated *I*_Na_ density back to a level comparable to the uninfected condition (Fig. 2C, Additional file 1: Table S1), consistent with the augmentation of Na_v_1.6-mediated *I*_Na_ density of CA1 neurons being mediated via altered TNF signaling. We next investigated if JAK2 activation upon TNFR1 stimulation contributed to the augmented Na_v_1.6-mediated *I*_Na_ density of CA1 pyramidal neurons induced by *P*. *chabaudi* infection by acutely treating slices from eCM mice with the JAK2 inhibitor fedratinib (Fig. 2B). Consistent with JAK2 functioning as a key node in our proposed signaling network, Fedratinib blocked the potentiation of Na_v_1.6-mediated *I*_Na_ density induced by *P*. *chabaudi* infection (Fig. 2C, Additional file 1: Table S1). Lastly in voltage-clamp mode, we tested the hypothesis that increased FGF14/Na_v_1.6 complex assembly induced by phosphorylation of FGF14^Y158^ mediated by TNFR1–JAK2 signaling was the determinant underlying augmented Na_v_1.6-mediated *I*_Na_ density observed in CA1 pyramidal neurons in slices from eCM mice. To this end, we treated brain slices from eCM mice with the FGF14 inhibitor ZL181 (Fig. 2B), which engages with the FGF14^Y158^ phosphorylation site [29, 32], and showed that treatment of slices from the eCM mice with ZL181 restored the Na_v_1.6-mediated *I*_Na_ density to a level comparable to the control condition (Fig. 2C, Additional file 1: Table S1).

Given that Na_v_1.6-mediated *I*_Na_ is a prominent determinant of intrinsic firing properties of CA1 pyramidal neurons [24], we next sought to determine if the changes in Na_v_1.6-mediated *I*_Na_ density induced by *P*. *chabaudi* infection would cause changes in action potential discharge of CA1 pyramidal neurons. To do so, we performed whole-cell current-clamp recordings in CA1 pyramidal neurons in slices from eCM mice (Fig. 2F, Additional file 1: Table S2). *P. chabaudi* infection induced an increase in the number of action potentials fired by CA1 pyramidal neurons over a range of injected current stimuli (Fig. 2G, Additional file 1: Table S2). Neither the infection nor the pharmacological inhibitors altered action potential kinetics (Fig. 2H, Additional file 1: Table S2). Similar to TNFR1 antagonism, JAK2 inhibition, and FGF14 inhibition blocking the augmentation of Na_v_1.6-mediated *I*_Na_ induced by *P*. *chabaudi* infection, R-7050, Fedratinib, and ZL181, respectively, also blocked the hyperexcitability phenotype and restored action potential discharge back to a level comparable to the uninfected condition (Fig. 2G, Additional file 1: Table S2). Collectively considered, these studies provide evidence for TNFR1–JAK2–FGF14–Na_v_1.6 as a novel signaling pathway that mediates neuronal hyperactivity in response to *P. chabaudi* infection.

### In vivo treatment with anti-TNF antibody mitigates neuronal hyperexcitability in eCM

**Fig. 3:**
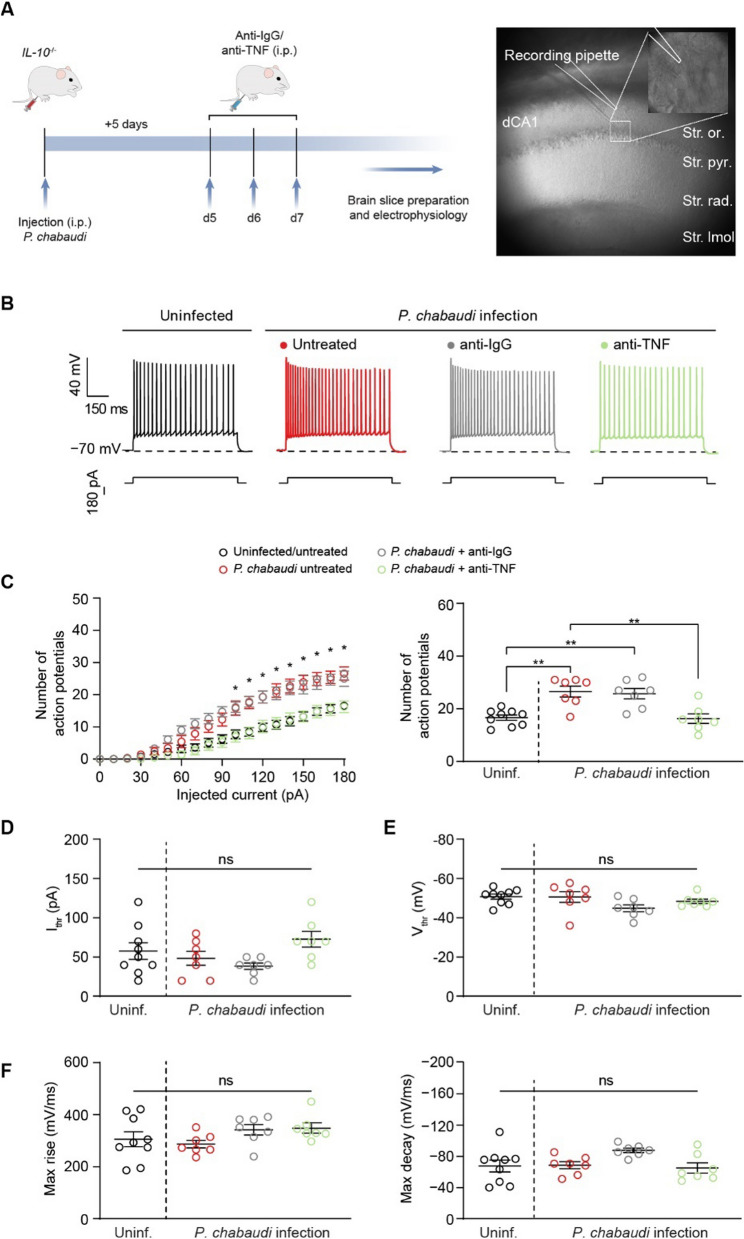
In vivo treatment with anti-TNF antibody mitigates neuronal hyperexcitability in eCM. **A** Left: timeline for infection, antibody treatment, and electrophysiological recordings. Right: representation of patch-clamp recording of CA1 pyramidal neuron. **B** Representative traces of action potentials fired by CA1 neurons in slices from the indicated experimental group at the 180-pA injected current step. **C** Number of action potentials fired by CA1 neurons over a range of injected current stimuli. **D**, **E** Comparison of the current (**D**) and voltage (**E**) thresholds for action potential initiation among the indicated experimental groups. **F** Comparison of action potential kinetics among the indicated experimental groups. Data are mean ± SEM (*n* = 7–9 neurons/group; slices from at least *N* = 3 mice per group). Significance was assessed using a one-way ANOVA with post hoc Tukey’s multiple comparisons test. In (**C**, right, **D**, **E**, and **F**): ns, not significant; **p < 0.01. In (**C**, left), * denotes current steps at which the number of action potentials fired by CA1 neurons of infected mice and infected mice treated with the isotype-matched control antibody is significantly greater (*p* is at least < 0.05) than the number of action potentials fired by CA1 neurons of uninfected mice and infected mice treated with the anti-TNF antibody

Neutralizing TNF prevents most of the symptoms in this model of hyperinflammatory experimental cerebral malaria, including gliosis, increased levels of fibrin(ogen) in the brain, behavioral changes, and mortality, without affecting parasitemia [20, 23]. Thus, we next sought to investigate whether the same in vivo treatment would mitigate CA1 neuron hyperexcitability induced by *P. chabaudi* infection. Infected mice received an intraperitoneal (IP) injection of either an anti-TNF antibody or isotype-control antibody days 5–7 p.i. On day 7 p.i., whole-cell current-clamp recordings in CA1 pyramidal neurons were performed (Fig. 3A). CA1 pyramidal neurons in slices from infected mice displayed augmented excitability compared to uninfected mice (Fig. 3B, Additional file 1: Table S3) over a range of injected current stimuli (Fig. 3C). In addition, neither infection nor antibody treatment affected the current (Fig. 3D) or voltage (Fig. 3E) thresholds for action potential initiation or action potential kinetics (Fig. 3F, Additional file 1: Table S3). Whereas administration of the isotype-control antibody did not alter effects of infection on CA1 neuron excitability (Fig. 3C), systemic in vivo neutralization of TNF reversed the effects of *P*. *chabaudi* infection on CA1 neuron excitability and restored the action potential discharge back to a level comparable to the control condition (Fig. 3C, Additional file 1: Table S3). Overall, these findings provide in vivo support for TNF-mediated changes in CA1 neuron excitability induced by *P. chabaudi* infection.

### In vivo genetic silencing of FGF14 mitigates hyperexcitability and altered plasticity of hippocampal neurons in eCM

**Fig. 4:**
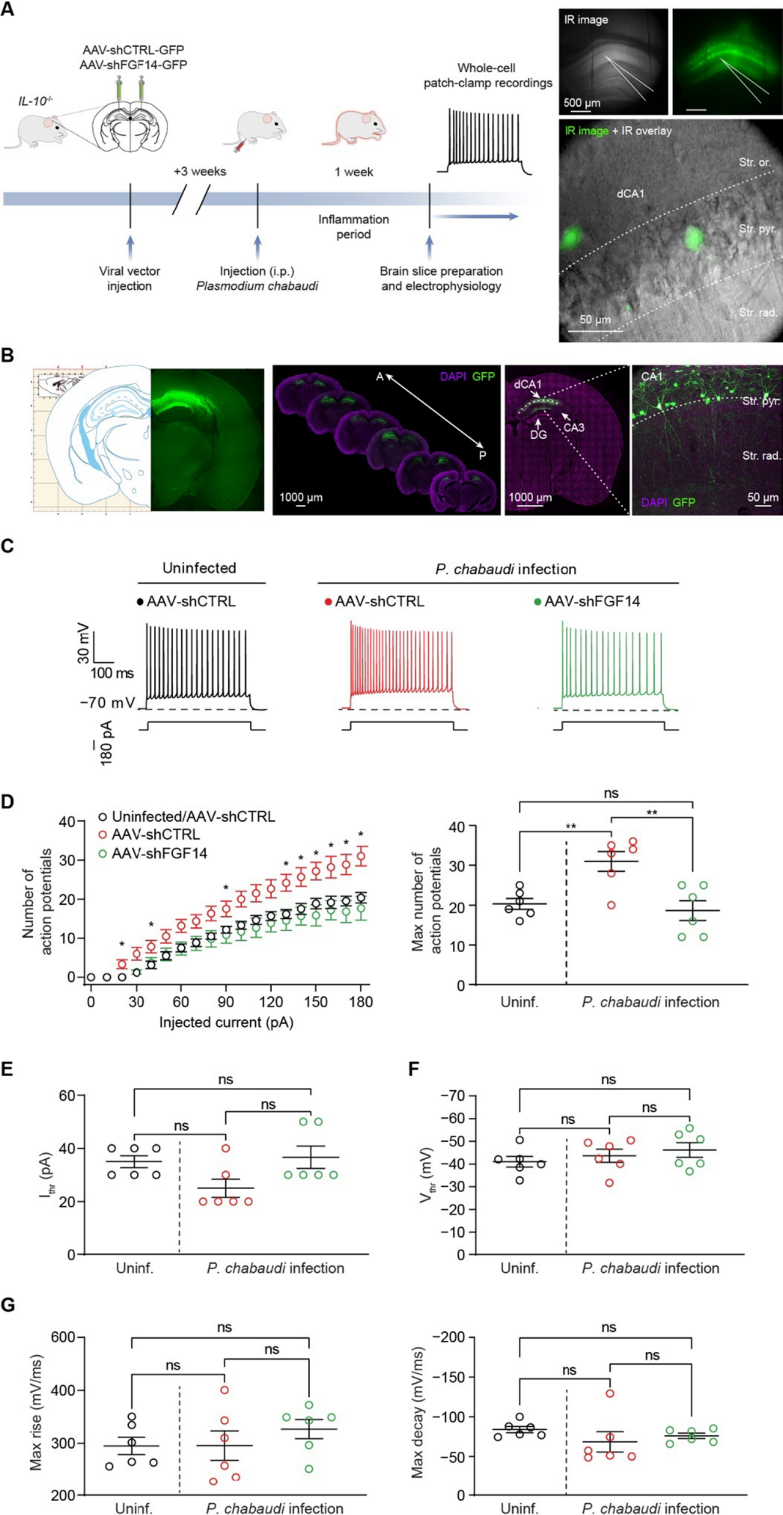
In vivo genetic silencing of FGF14 mitigates hyperexcitability of CA1 neurons caused by eCM. **A** Left: timeline for stereotaxic surgery, *P*. *chabaudi* infection, and electrophysiological recordings. Right: representation of whole-cell patch-clamp recording in GFP.^+^ CA1 pyramidal neuron. **B** Confirmation of accurate vector placement in CA1 region of hippocampus. **C** Representative traces of action potentials fired by CA1 neurons in slices from the indicated experimental groups at the 180-pA injected current step. **D** Left: number of action potentials fired by CA1 neurons over a range of injected current stimuli. Right: comparison of the maximum number of action potentials fired by CA1 neurons among the indicated experimental groups. **E**, **F** Comparison of the current threshold (I_thr_; **E**) and voltage threshold (V_thr_; **F**) for CA1 neuron action potential initiation among the indicated experimental groups. **G** Comparison of action potential kinetics among the indicated experimental groups. Data are mean ± SEM (*n* = 6 neurons/group; slices from *N* = 3 mice per group). Statistical significance was assessed using a one-way ANOVA with post hoc Tukey’s multiple comparisons test. In (**D**, right–**G**): *ns* not significant; **p < 0.01. In (**D**, left), * denotes current steps at which the number of action potentials fired by CA1 neurons from infected mice injected with AAV-shCTRL is significantly greater (*p* is at least < 0.05) than the number of action potentials fired by CA1 neurons from uninfected mice injected with AAV-shCTRL and infected mice injected with AAV-shFGF14

After confirming FGF14’s contribution to hyperexcitability in eCM through TNFR1–JAK2 signaling, we sought to determine whether genetic silencing FGF14 could prevent these phenotypes. A short hairpin targeting *Fgf14* was designed and expressed in a GFP reporter construct (AAV-shFGF14) and packaged into Adeno Associated Virus 2, a viral serotype with selective neuronal tropism [61]. AAV-shFGF14 or a control vector (referred to as AAV-shCTRL[44]) were stereotaxically injected into the CA1 region of the mouse hippocampus. At least 3 weeks after injection, whole-cell current-clamp recordings were performed in slices from uninfected *IL-10*^−/−^ mice injected with either AAV-shCTRL or AAV-shFGF14 to functionally validate the vector. Crucially, visually identified GFP^+^ CA1 neurons expressing AAV-shFGF14 displayed intrinsic firing properties comparable to CA1 neurons from *Fgf14*^*−/−*^ mice (Additional file 1: Figure S2A) [27], including a reduction in firing over a range of injected current stimuli (Additional file 1: Figure S2B, C) coupled with an increase in current threshold (Additional file 1: Figure S2D). Also consistent with previously reported regulatory effects of FGF14 on CA1 neuron activity [27], knocking down *Fgf14* did not affect other active (Additional file 1: Figures S2E-G) or passive (Additional file 1: Figure S2H, I) electrical properties of CA1 neurons. Next, *IL-10 *^*−/−*^ mice were injected with either AAV-shCTRL or AAV-shFGF14 into the CA1 region and infected with *P. chabaudi* 3 weeks after AAV injection. Whole-cell current-clamp recordings were subsequently performed on day 7 p.i. in CA1 pyramidal neurons (Fig. 4A, B) in slices from uninfected mice injected with AAV-shCTRL, or eCM mice injected with AAV-shCTRL or AAV-shFGF14 (Fig. 4C). eCM mice treated with AAV-shCTRL displayed increased action potential firing over a range of injected current stimuli, as well as an increase in the maximum number of action potentials, compared to uninfected mice treated with AAV-shCTRL (Fig. 4D, Additional file 1: Table S4). Neither the infection nor the AAV treatments exerted effects on the current (Fig. 4E) or voltage (Fig. 4F) thresholds for action potential initiation or action potential kinetics (Fig. 4G, Additional file 1: Table S4). Compared to AAV-shCTRL treatment, AAV-shFGF14 treatment mitigated the hyperexcitability phenotype caused by *P*. *chabaudi* infection and restored action potential firing back to a level comparable to the uninfected control condition (Fig. 4D, Additional file 1: Table S4), which demonstrates that in vivo modulation of the TNFR1–JAK2–FGF14–Na_v_1.6 signaling network at the level of FGF14 is sufficient to block the perturbations in neuronal excitability caused by infection.

**Fig. 5:**
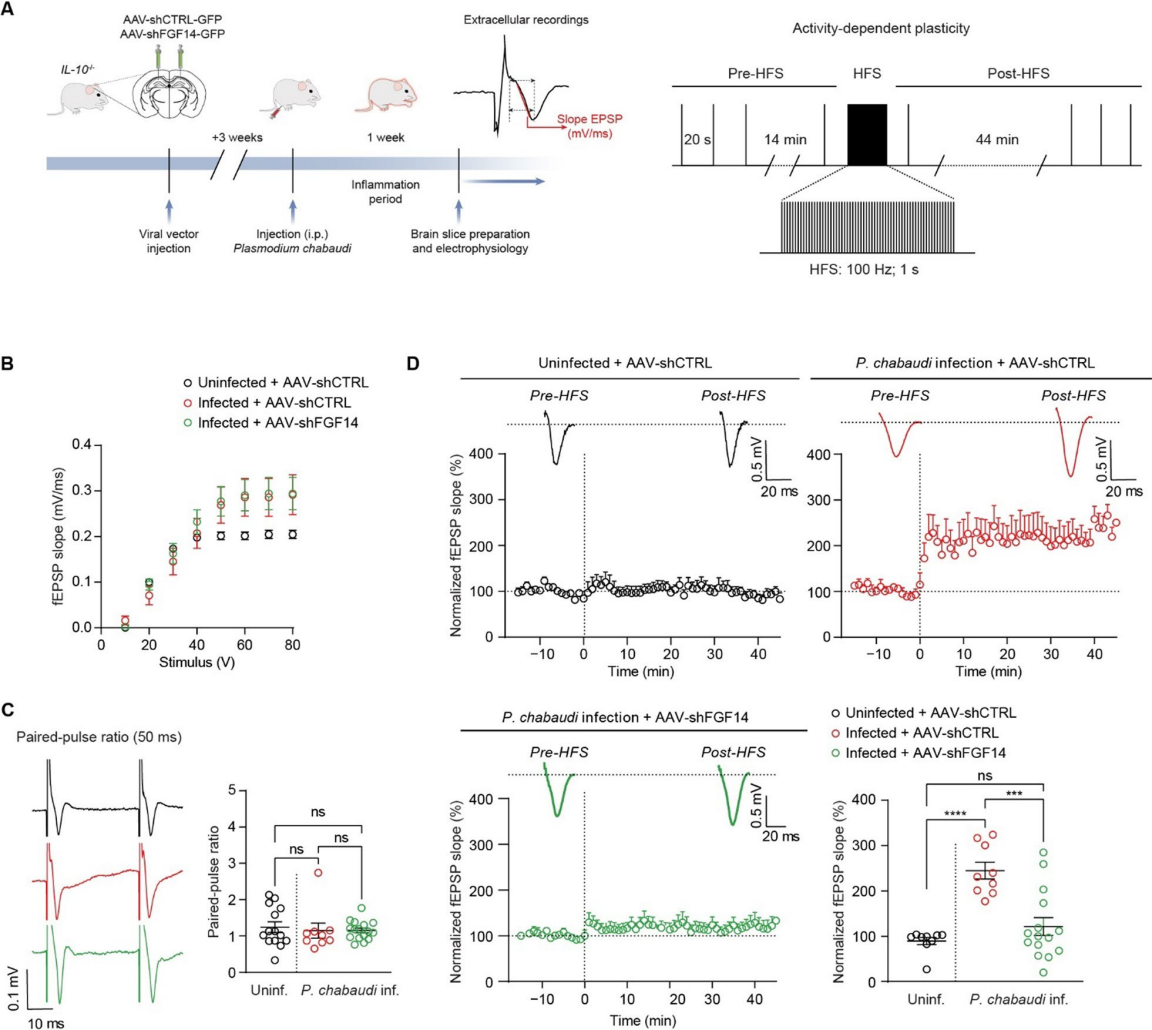
In vivo genetic silencing of FGF14 mitigates altered plasticity of hippocampal neurons in eCM. **A** Left: timeline of stereotaxic surgery, *P*. *chabaudi* infection, and field recordings. Right: schematic of HFS protocol used in **D**. **B** fEPSP slope plotted as a function of stimulation intensity to assess basic synaptic responses in slices from the indicated experimental groups. **C** Comparison of PPF among the indicated experimental groups. **D** Comparison of activity-dependent plasticity among the indicated experimental groups. Data are mean ± SEM (*n* = 9–14 slices/group; slices from *N* = 4 mice per group). Statistical significance was assessed using a one-way ANOVA with post hoc Tukey’s multiple comparisons test. In (**C**, right and **D**, bottom right): *ns* not significant; ***p < 0.001; ****p < 0.0001

Seizures occur in children with cerebral malaria and impact the likelihood of survival [62]. Susceptibility to seizures is influenced by hippocampal network excitability, which is governed by both intrinsic excitability of neurons and synaptic efficacy [63, 64]. Having shown that intrinsic excitability of CA1 pyramidal neurons is increased in eCM by TNFR1 signaling through FGF14 phosphorylation, we next sought to investigate if synaptic efficacy is altered through FGF14 to further increase hippocampal network excitability. To do so, we conducted field potential recordings in slices from infected mice that were stereotaxically injected with either AAV-shCTRL or AAV-shFGF14 in the CA1 region of the hippocampus. Similar to patch-clamp studies, at least 3 weeks after AAV injection, mice were infected with *P. chabaudi*, and recordings were performed on day 7 p.i. (Fig. 5A). We first characterized basic synaptic transmission at the primary excitatory inputs to CA1, namely the CA3-CA1 synaptic inputs. Basic synaptic responses (measured as the field excitatory post-synaptic potential slope; fEPSP slope) from each group were similar across a range of stimulation intensities (Fig. 5B). Although there was a trend toward an increased fEPSP slope at stimulation intensities over 40 V in the infected groups, this did not reach statistical significance. Evaluation of synaptic responses using a paired-pulse facilitation (PPF) protocol, indicative of pre-synaptic release probability, also showed no differences (Fig. 5C). Next, we investigated the effects of *P*. *chabaudi* infection on CA3-CA1 synapses using a high-frequency stimulation (HFS) protocol (Fig. 5A). Using a 100 Hz/sec burst stimulation, we observed that CA3-CA1 synaptic strength after HFS was significantly increased in slices from eCM mice treated with AAV-shCTRL compared to uninfected mice treated with AAV-shCTRL (Fig. 5D). Notably, AAV-mediated silencing of FGF14 in CA1 mitigated the augmented CA3-CA1 synaptic strength in response to HFS induced by *P*. *chabaudi* and restored it to a level comparable to the uninfected AAV-shCTRL condition (Fig. 5D). Overall, these results, coupled with our patch-clamp data, demonstrate that hippocampal network excitability is increased in hyperinflammatory *P*. *chabaudi* infection by increasing the intrinsic excitability of neurons and also increasing synaptic efficacy through signaling involving FGF14, which could underlie increased susceptibility to seizures during cerebral malaria.

### In vivo genetic silencing of FGF14 in the hippocampus ameliorates sickness behavior and controls thermoregulation in eCM

**Fig. 6:**
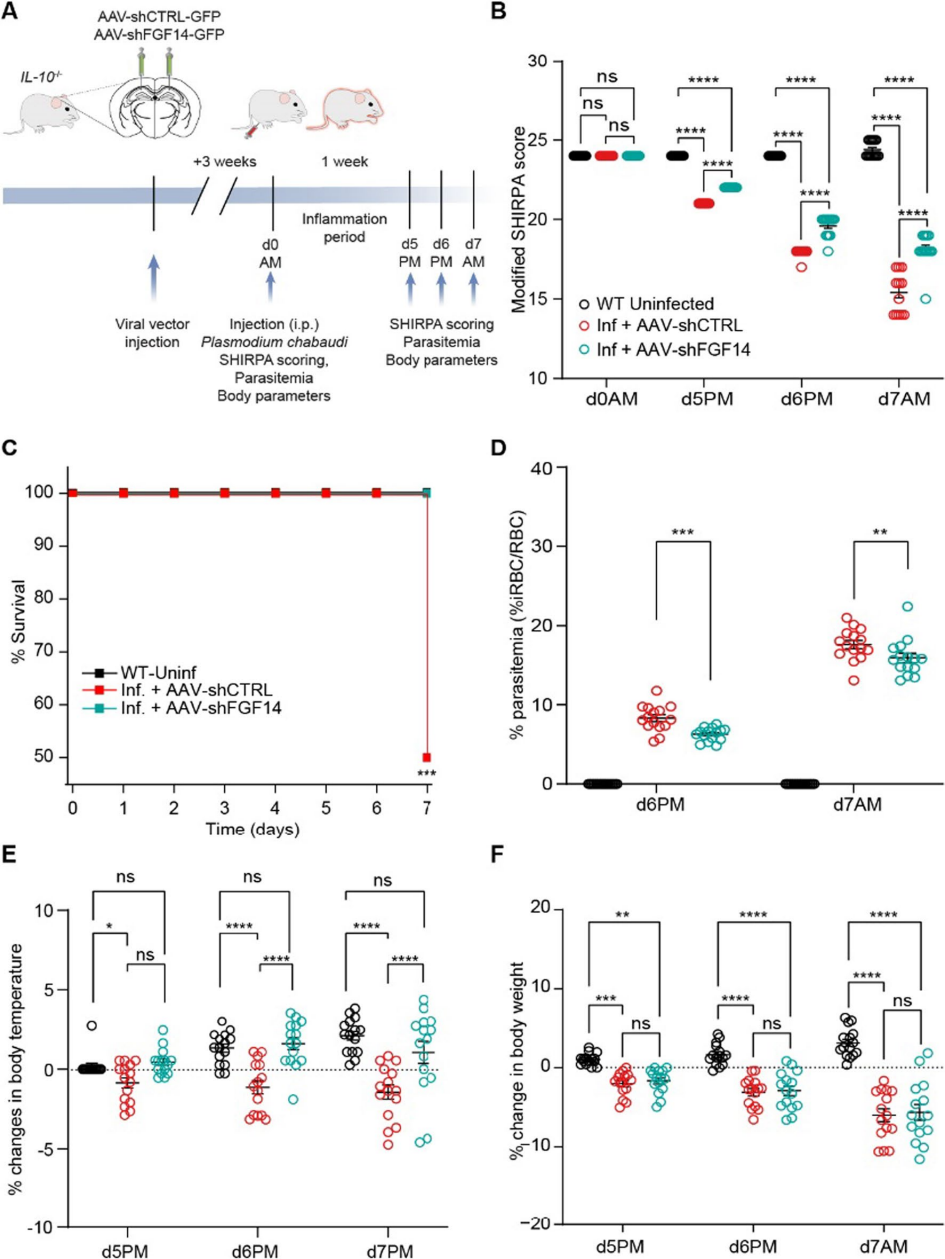
In vivo genetic silencing of FGF14 in the hippocampus ameliorates sickness behavior and controls thermoregulation in eCM. **A** Timeline for evaluation of sickness behavior and other consequences of *P*. *chabaudi* infection. eCM was measured by **B** modified SHIRPA behavioral scores, **C** predicted survival based upon SHIRPA scores day 7 p.i., **D** parasite load (%iRBC/RBC), **E** hypothermia, and **F** weight loss for mice of the indicated groups. Data shown as mean ± SEM (*n* = 15 mice/group). In **B**–**F**, statistical significance was determined via a 2-way ANOVA with post hoc Tukey’s multiple comparisons test: *ns* not significant; *p < 0.05; **p < 0.01; ***p < 0.001; ****p < 0.0001

Having demonstrated that in vivo modulation of our proposed signaling network at the level of FGF14 is sufficient to mitigate hippocampal perturbations induced by *P*. *chabaudi* infection, we next sought to investigate whether the same manipulation had any effect on sickness behavior in these mice. To that end, *IL-10*^*−/−*^ mice were stereotaxically injected with AAV-shCTRL or AAV-shFGF14 into the CA1 region of the hippocampus. 3 weeks after injection, mice were infected with *P. chabaudi*. Following infection, mice were assessed daily using a modified version of the SmithKline Beecham, Harwell, Imperial College, Royal London Hospital Phenotype Assessment (SHIRPA) Protocol previously shown to capture behavioral symptoms of cerebral malaria in this model [22] (Fig. 6A). A modified SHIRPA assessment includes nine of the forty semi-quantitative SHIRPA tests that best correlate with mortality in *P. chabaudi* infection of *IL-10*^−/−^ mice [23]. The selected tests also correspond well with published behavioral assays for experimental cerebral malaria in *P. berghei* ANKA [65]. The assessment includes tests of general health, sensory function, baseline behaviors, and neurological reflexes, with a score below 16 before day 9 being predictive of decreased survival [22, 23, 50]. Indicative of the onset of eCM, the modified SHIRPA score of both groups of infected mice decreased compared to uninfected mice by the evening of the 5th day of infection (Fig. 6B). At this time, there was already a significant difference between the SHIRPA scores of infected groups receiving AAV-shCTRL or AAV-shFGF14 (Fig. 6B), which indicates that genetic silencing of FGF14 in CA1 neurons caused a significant decrease in the behavioral symptoms of hyperinflammatory eCM. There was also a significant improvement in the survival curve in infected mice treated with AAV-shFGF14 compared to infected mice treated AAV-shCTRL (Fig. 6C). In addition to improving behavioral measures of eCM, infected mice treated with AAV-shFGF14 in the CA1 region of the hippocampus displayed significantly reduced parasitemia compared to infected mice treated with AAV-shCTRL (Fig. 6D). It was possible that parasitemia is reduced due to changes in systemic cytokines; however, this was not found to be the case. Neither TNF (Additional file 1: Figure S5A) nor IFN-γ (Additional file 1: Figure S5B) concentrations were changed in the hippocampi or sera of mice in the two infected groups, suggesting that the change in parasitemia is not downstream of systemic cytokine levels. To investigate if silencing of FGF14 in CA1 neurons could prevent the sickness behavior seen in severe malaria, body temperature and weight loss of mice were assessed. As previously reported, *IL-10*^*−/−*^ mice infected with *P. chabaudi* develop hypothermia [20]. However, there was no hypothermia in infected mice treated with AAV-shFGF14, whereas infected mice treated with AAV-shCTRL displayed levels of hypothermia consistent with untreated *P. chabaudi* infection (Fig. 6E). This protective effect of genetic silencing of FGF14 in CA1 neurons on body temperature, however, was not generalized*,* as weight loss from cachexia was not different between the two groups of infected mice (Fig. 6F), indicating that silencing FGF14 in the CA1 region of the hippocampus in the context of eCM leads to a specific effect on thermoregulation.

## Discussion

Using a combination of cell-based assays, high-throughput screening, brain slice electrophysiology, in vivo genetic silencing, and studies of sickness behaviors, we have demonstrated a TNFR1–JAK2–FGF14–Na_v_1.6 signaling network in neurons and established its physiological relevance to the neuro-immune axis. Elucidation of this pathway also mechanistically links elevated levels of TNF in the hippocampus and neurological dysfunction caused by *Plasmodium spp*. infection. Both host and parasite factors contribute to severity in cerebral malaria. In order to specifically identify neural mechanisms of eCM due to inflammatory cytokines, we used infection of *IL-10*^*−/−*^ mice with *P. chabaudi* as an experimental cerebral malaria model, which is a TNF-dependent model of eCM, where a mildly virulent parasite is made more virulent by reduced immuno-regulation. This allows us to conclude that inflammatory cytokines, as distinct from parasite virulence factors, can directly affect neuronal function during cerebral malaria. While *P. berghei* ANKA infection of C57BL/6J wild-type mice is a more commonly used combination to study eCM, severity in that model relies on parasite virulence, while immune regulation is less well understood. Both models include severe neuroinflammation; however, *P. berghei* ANKA is driven by parasite factors in addition to host factors. Neurological sequelae observed in human cerebral malaria include epilepsy [66] and cognitive impairment [67]. Of relevance then, gain-of-function mutations in the *Scn8a* gene, which encodes the Na_v_1.6 channel isoform, lead to the development of epilepsy and intellectual disabilities [68]. Thus, our finding that hyperinflammatory eCM enhances the activity of the Na_v_1.6 channel suggests potential molecular mechanisms contributing to seizures and cognitive impairments induced by cerebral malaria.

TNF binding causes TNFR1 to trimerize and form complexes with tyrosine kinases, including c-Src and JAK2 [53]. These complexes auto-phosphorylate, which initiates cell signaling cascades, activates transcription factors [53], and alters gene expression [69–73]. Related to initiation of TNFR cell signaling cascades, TNFR1/JAK2 activates Akt, JNK and p38 MAPK, while c-Src only activates Akt [53]. Our studies reveal that a phosphorylation cascade initiated by TNFR1 and mediated by JAK2 increases complex assembly of FGF14 with the Na_v_1.6 channel. This is evidenced by pharmacological inhibition of JAK2 and mutation of FGF14^Y158^ (where JAK2 is reported to phosphorylate FGF14 [32]) being sufficient to negate the effect of TNF on FGF14/Na_v_1.6 complex assembly and channel function. This finding demonstrates that the regulation of FGF14/Na_v_1.6 complex assembly by JAK2 and TNFR1 is direct, and not dependent upon activation of Akt, JNK, or p38 MAPK. Whereas TNFR1 stimulation causes JAK2 to directly promote increased FGF14/Na_v_1.6 complex assembly, it does not affect FGF14/FGF14 dimer assembly. This finding is notable because our previous study found that under basal conditions, JAK2 promotes dissociation of the dimer [32], which, coupled with the findings of the present investigation, indicates that TNF signaling alters the function of JAK2 in terms of regulating the monomeric:dimeric stoichiometry of FGF14. In neurons, such specialized and rapid signal transduction processes could enable a prompt cellular response to TNFR1 activation under inflammatory conditions.

Our cell signaling studies expand upon current knowledge of how TNFR1 signals to control neuronal function. While Pincheria et al. [53] previously showed that TNF binding to TNFR1 can increase TNFR1/JAK2 complex formation and JAK2 signaling, the downstream signaling network, and use of JAK2 versus NF-ΚB, was not well defined in neurons. We showed in previous reports that TNF increases FGF14/Na_v_1.6 complex assembly [36], and that phosphorylation of FGF14^Y158^ by JAK2 increases FGF14/Na_v_1.6 complex assembly [32], but the signaling pathway driving this mechanism in neurons was underdefined. In our current study, we integrate all these findings in neurons and support the critical function of this neuroinflammatory pathway converging on FGF14 in vivo. Additional in vitro inhibition studies here provide evidence that TNFR1 signaling leads to increased tyrosine kinase activity of JAK2, which leads to increased phosphorylation of FGF14^Y158^ and a resultant increase in FGF14/Na_v_1.6 complex assembly. Another important finding is that whereas activation of the TNFR1/JAK2 pathway leads to increased phosphorylation of FGF14^Y158^ and a resultant increase in FGF14/Na_v_1.6 complex assembly, NF-ΚB appears to be less involved in our signaling network, as the IKK inhibitor CAY10576 did not block the increase in FGF14/Na_v_1.6 complex assembly induced by TNF treatment. Overall, these findings provide novel insights of how the TNFR1/JAK2 branch of TNF signaling functions to regulate neuronal function in response to neuroinflammatory conditions.

While TNF’s regulatory effects on Na_v_ channel function in peripheral sensory neurons have been well-studied [12, 74–77], such effects have received some, although less, attention in neurons of the brain [78]. For example, peripherally in dorsal root ganglia, TNF has been shown to increase Na_v_1.3 [75, 77], Na_v_1.7 [76], and Na_v_1.8 [75] channel activity; and in retinal ganglion cells, TNF was shown to increase Na_v_1.6, but not Na_v_1.1 or Na_v_1.2, channel activity [12]. Our finding that CA1 pyramidal neurons in slices from eCM mice displayed augmented Na_v_1.6-mediated *I*_Na_ density is similar to the finding of Cheng et al*.* [12] showing that TNF increased Na_v_1.6 channel activity in retinal ganglion cells. Crucially, we extend their findings by defining signaling pathways involved and molecular mechanisms of regulation of channel activity by TNF. Acute (ex vivo) pharmacological inhibition of each signaling molecule in the TNFR1–JAK2–FGF14 pathway was sufficient to block the effect of infection on Na_v_1.6-mediated *I*_Na_ density. Increases in *I*_Na_ density can result either from more channels being trafficked to the plasma membrane or through changes in biophysical properties of the channel that alter open channel probability. Given that the voltage-dependences of activation and inactivation of Na_v_1.6-mediated *I*_Na_ were not affected by the infection or sensitive to the pharmacological manipulations tested, the effect of TNF on *I*_Na_ through the TNFR1–JAK2–FGF14–Na_v_1.6 signaling network is likely mediated by increased trafficking of channels to the plasma membrane. Additionally, given that 1 h treatment of slices from eCM mice with inhibitors of signaling molecules in the pathway was able to mitigate the augmented *I*_Na_ density caused by *P*. *chabaudi* infection, regulatory effects of TNFR1–JAK2–FGF14 signaling on Na_v_1.6 channel function likely occur through modulation of existing pools of channels, as opposed to causing transcriptional upregulation of channel activity.

Consistent with *P*. *chabaudi* infection causing an increase in *I*_Na_ density of CA1 pyramidal neurons through the TNFR1–JAK2–FGF14–Na_v_1.6 signaling network, which we posit occurs through TNF inducing increased FGF14/Na_v_1.6 complex assembly leading to increased trafficking of Na_v_1.6 channels to the plasma membrane, the infection correspondingly increased the intrinsic excitability of hippocampal neurons. Also consistent with the voltage-clamp studies, acute (ex vivo) pharmacological inhibition of all of the proposed signaling molecules in the TNFR1–JAK2–FGF14–Na_v_1.6 pathway ameliorated the hyperexcitability phenotype induced by infection, demonstrating that disruption of the signaling network is sufficient to block features of eCM related to neuronal excitability. Expanding on these findings, we show that chronic (in vivo) manipulation of the signaling network via systemic administration of a TNF neutralizing antibody is sufficient to mitigate the hyperexcitability phenotype caused by infection. This treatment inhibits vascular permeability as well as patent vascular hemorrhage in *P. chabaudi* infected animals, and eliminates all measurable behavioral changes associated with hyperinflammatory eCM [20, 79]. However, the effects of TNF within the brain parenchyma, particularly directly on neurons, had not previously been investigated. Grau et al. [80] found that levels of TNF mRNA were elevated in the brain of eCM susceptible but not resistant mice, while TNF expression was the same in the spleens of mice with or without *P. berghei* ANKA-induced eCM, highlighting the importance of local TNF in the brain for the manifestations of cerebral malaria. mRNA and protein levels of TNF in the brain are elevated at day 3 and day 5 p.i., respectively, in *P*. *berghei* ANKA-induced eCM [81]. As mice begin to show symptoms of cerebral malaria, there is a significant increase in the number of cells with morphological characteristics of microglia and astrocytes expressing TNF [81], which along with systemic TNF, induces permeability of the blood brain barrier (BBB) [82, 83]. Given the increased permeability of the BBB observed in the eCM model used in the present investigation [79], it is likely that the anti-TNF antibody we employed is able to reach the brain and neutralize elevated levels of TNF produced by microglia and astrocytes. Through this mechanism, coupled with systemic neutralization, the anti-TNF antibody is able to mitigate the hyperexcitability phenotype induced by *P*. *chabaudi* infection.

Next, we show that downstream manipulation of the signaling network via in vivo genetic silencing of FGF14 in the hippocampus is similarly sufficient to mitigate the increased excitability of CA1 pyramidal neurons observed in eCM. Expanding upon these cellular findings, we further show that at the network level, in vivo genetic silencing of FGF14 in the CA1 region blocks changes in hippocampal synaptic plasticity observed in eCM. Crucially, previous studies have found that glial derived TNF causes a rapid increase in the number of surface-localized, synaptic AMPA receptors in hippocampal neurons, which leads to an increase in synaptic strength [84]. Consistent with this, TNF has been shown to increase the ratio of AMPA receptor- to NMDA receptor-mediated synaptic currents [9] and to increase basal synaptic transmission [85]. However, acute (ex vivo) treatment of slices with recombinant TNF protein has been shown to either have no effect on long-term potentiation (LTP) [9] or to inhibit LTP [85–87], depending upon the concentration of protein used, duration of pre-treatment, and stimulation protocol. In contrast to these findings, Wall et al. [88] reported that after acute hypoxia, TNF treatment did increase LTP. Pertinent to the present investigation, a prominent component of the pathogenesis of cerebral malaria is increased coagulation, slowed blood flow, and damaged vascular endothelium, likely resulting in brain hypoxia [89]. Given this, the increase in CA3-CA1 synaptic strength after HFS observed in eCM may result from elevated levels of TNF in the hippocampus coupled with hypoxia caused by *P*. *chabaudi* infection. While the precise mechanism through which knockdown of FGF14 inhibits the infection-induced increase in CA3-CA1 synaptic strength after HFS remains unclear, previous studies have demonstrated a prominent role of Na_v_ channels in regulating amplification of synaptic responses under basal conditions [90, 91] and in response to HFS [92]. Thus, it is possible that knockdown of FGF14 in CA1 mitigates the increased CA3-CA1 synaptic responses in response to HFS observed in eCM by reducing Na_v_ channel activity [92]. Furthermore, JAK2 is involved in plasticity induction [93] and synapse stability [94], potentially linking it to the enhanced synaptic responses observed in eCM due to TNF activation. In addition, whereas *P*. *chabaudi* infection increases CA3-CA1 synaptic strength after HFS primarily through post-synaptic changes, previous studies have found that genetic deletion of FGF14 affects pre-synaptic mechanisms of LTP [95], which suggests that neuroinflammatory conditions could alter FGF14’s function in terms of regulating synaptic activity.

Although the exact mechanism by which knocking down FGF14 in CA1 prevents hypothermia induced by *P*. *chabaudi* and reduces parasitemia throughout the course of infection is unclear, a likely candidate is through modulation of hippocampal-hypothalamic connectivity, the latter of which is centrally involved in the hypothalamic–pituitary–adrenal (HPA axis) [96] and serves as a canonical regulator of thermoregulation [97, 98]. Pertinent to thermoregulation, previous studies have demonstrated that optogenetic activation of GABAergic neurons in the ventral lateral preoptic nucleus (vLPO) of the hypothalamus reduces body temperature by disinhibition of the dorsomedial hypothalamus [97]. In eCM, increased glutamatergic hippocampal output caused by CA1 pyramidal neuron hyperexcitability may lead to elevated glutamate levels in the hypothalamus, potentially causing overactivation of GABAergic neurons in the vLPO and resulting in hypothermia. Silencing FGF14 prevents CA1 hippocampal hyperexcitability, which reduces the hippocampal output and could inhibit excessive glutamate release onto the hypothalamus, which could thereby prevent hypothermia. Related to the reduction in parasitemia throughout the course of infection, elevated hypothalamic glutamate levels have been linked to increased activation of the HPA axis [99], leading to enhanced production and release of glucocorticoids (GC) by the adrenal cortex. Silencing FGF14 in CA1, which may reduce glutamate release onto the hypothalamus, could decrease HPA axis activation, thereby lowering GC levels in the bloodstream. This could potentially impact macrophage differentiation towards a pro-inflammatory phenotype and reduce parasitemia [100]. However, further research on the role of FGF14 in controlling the hippocampal–hypothalamic axis and GC release in eCM is needed to validate these hypotheses.

## Conclusions

Overall, our findings have implications not only for cerebral malaria and neuroinfectious diseases, but also for other neuroinflammatory conditions. TNF plays a role in neurodegenerative disorders like Alzheimer's disease [101, 102] and neuropsychiatric disorders [103]. The connection between increased TNF signaling and hyperexcitability in the CA1 region through the TNFR1-JAK2-FGF14-Na_v_1.6 signaling network could provide a molecular basis for aspects of all these conditions.

### Supplementary Information

**Additional file 1: Figure S1.** In vitro validation of AAV-shFGF14. qPCR quantification of Fgf14 mRNA in HEK293 cells co-transfected with a plasmid overexpressing FGF14 and shCTRL (positive control) or HEK293 cells co-transfected with a plasmid overexpressing FGF14 and one of five short hairpins targeting the Fgf14 coding region. **Figure S2.** Ex vivo validation of the AAV-shFGF14 vector. (A) Representative traces of action potentials fired by CA1 neurons expressing the AAV-shCTRL (black) or AAV-shFGF14 (blue) construct in response to the depicted current clamp protocol.(B) Number of action potentials fired by CA1 neurons belonging to the experimental groups described in (A) over a range of injected current stimuli. (C) Comparison of the maximum number of action potentials fired by CA1 neurons belonging to the indicated experimental groups. (D,E) Comparison of the current threshold (Ithr) (D) and voltage threshold (Vthr) (E) for the action potential initiation of CA1 neurons belonging to the indicated experimental groups. (F,G) Comparison of the maximum rise (F) and maximum decay (G) of action potentials fired by CA1 neurons belonging to the indicated experimental groups. (H,I) Comparison of the input resistance and resting membrane potential (RMP) of CA1 neurons belonging to the indicated experimental groups. Data are mean ± SEM (n = 3–10 cells/group; slices from N = 2–3 mice per group). Significance was assessed using a Student’s t-test. In (C-I): ns, not significant; **, p < 0.01; ***, p < 0.001. In (B), * denotes current steps at which the number of action potentials fired by CA1 neurons expressing AAV-shFGF14 is significantly lower (p is at least < 0.05) than CA1 neurons expressing AAV-shCTRL. **Figure S3.** Summary of high-throughput screening of Broad Institute Collection against the FGF14:Nav1.6 complex with and without TNF stimulation. (A) Effects of 320 compounds from the Broad Institute Collection on FGF14:Nav1.6 complex assembly with and without TNF treatment. The compound assigned to each number is listed in Table S5. (B) Comparison of three JAK2 inhibitors (Fedratinib, Momelotinib, and NVP-BSK805) and one IKK inhibitor (CAY10576) on FGF14:Nav1.6 complex assembly with and without TNF stimulation. **Figure S4.** Presence of TNF in hippocampi of IL-10-/- mice infected with P. chabaudi on day 7 post-infection. Left: full, uncropped immunoblot of TNF from hippocampal lysate. Right: full, uncropped immunoblot of B-actin from hippocampal lysate. **Table S1.** P. chabaudi infection increases Nav1.6-mediated INa through TNFR1–JAK2 signaling. **Table S2.** P. chabaudi infection increases CA1 pyramidal neuron excitability through TNFR1–JAK2 signaling. **Table S3.** In vivo neutralization of TNF mitigates the hyperexcitability phenotype induced by P. chabaudi infection. **Table S4.** In vivo genetic silencing of FGF14 is sufficient to block hyperexcitability of CA1 pyramidal neurons caused by P. chabaudi infection. **Figure S5.** Genetic silencing of FGF14 in CA1 neurons does not affect TNF or IFN-γ production. (A) Comparison of protein levels of TNF between the indicated experimental groups in the hippocampus and serum. (B) Comparison of protein levels of IFN-γ between the indicated experimental groups in the hippocampus and serum. Data are mean ± SEM (n = 6–8 replicates per group). Statistical significance was assessed using a Student’s t-test: ns, not significant. **Table S5.** Compound ID for data shown in Figure S1. List identifying the compound name that corresponds to the compound numbers shown in Figure S1.

## Data Availability

All data generated or analyzed during this study are included in this published article.

## Abbreviations

TNFR1: Tumor necrosis factor receptor 1
FGF14: Fibroblast growth factor 14
TNF: Tumor necrosis factor
eCM: Experimental cerebral malaria
LCA: Split-luciferase complementation assay
Na_v_1.6: Voltage-gated Na^+^ channel isoform 1.6
JAK2: Janus kinase 2
IL-10: Interleukin-10
CTD: C-terminal domain
HTS: High-throughput screening
p.i.: Post-infection
I_Na_: Transient sodium current
IP: Intraperitoneal
AAV: Adeno-associated virus
fEPSP: Field excitatory post-synaptic potential
PPF: Paired-pulse facilitation
HFS: High-frequency stimulation
SHIRPA: SmithKline Beecham, Harwell, Imperial College, Royal London Hospital Phenotype Assessment
iRBC: Infected red blood cell
IFNγ: Interferon gamma
BBB: Blood brain barrier
LTP: Long-term potentiation
HPA axis: Hypothalamic–pituitary–adrenal axis
vLPO: Ventral lateral preoptic nucleus of the hypothalamus
GC: Glucocorticoids
