# Supplementary material for: TNFR1 signaling converging on FGF14 controls neuronal hyperactivity and sickness behavior in experimental cerebral malaria
Source: J Neuroinflammation. 2023 Dec 19;20:306. doi: 10.1186/s12974-023-02992-7 (PMC10729485; doi:10.1186/s12974-023-02992-7)
Supplement: Supplementary file 1 — Additional file 1: Figure S1. In vitro validation of AAV-shFGF14. qPCR quantification of Fgf14 mRNA in HEK293 cells co-transfected with a plasmid overexpressing FGF14 and shCTRL (positive control) or HEK293 cells co-transfected with a plasmid overexpressing FGF14 and one of five short hairpins targeting the Fgf14 coding region. Figure S2. Ex vivo validation of the AAV-shFGF14 vector. (A) Representative traces of action potentials fired by CA1 neurons expressing the AAV-shCTRL (black) or AAV-shFGF14 (blue) construct in response to the depicted current clamp protocol.(B) Number of action potentials fired by CA1 neurons belonging to the experimental groups described in (A) over a range of injected current stimuli. (C) Comparison of the maximum number of action potentials fired by CA1 neurons belonging to the indicated experimental groups. (D,E) Comparison of the current threshold (Ithr) (D) and voltage threshold (Vthr) (E) for the action potential initiation of CA1 neurons belonging to the indicated experimental groups. (F,G) Comparison of the maximum rise (F) and maximum decay (G) of action potentials fired by CA1 neurons belonging to the indicated experimental groups. (H,I) Comparison of the input resistance and resting membrane potential (RMP) of CA1 neurons belonging to the indicated experimental groups. Data are mean ± SEM (n = 3–10 cells/group; slices from N = 2–3 mice per group). Significance was assessed using a Student’s t-test. In (C-I): ns, not significant; **, p < 0.01; ***, p < 0.001. In (B), * denotes current steps at which the number of action potentials fired by CA1 neurons expressing AAV-shFGF14 is significantly lower (p is at least < 0.05) than CA1 neurons expressing AAV-shCTRL. Figure S3. Summary of high-throughput screening of Broad Institute Collection against the FGF14:Nav1.6 complex with and without TNF stimulation. (A) Effects of 320 compounds from the Broad Institute Collection on FGF14:Nav1.6 complex assembly with and without TNF treatment. Th [file 12974_2023_2992_MOESM1_ESM.docx]

**Additional file Information**

**TNFR1 signaling converging on FGF14 controls neuronal hyperactivity and sickness behavior in experimental cerebral malaria**

Nolan M. Dvorak, Nadia D. Domingo, Cynthia M. Tapia, Paul A. Wadsworth, Mate Marosi, Yosef Avchalumov, Chanida Fongsaran, Leandra Koff, Jessica Di Re, Catherine M. Sampson, Timothy J. Baumgartner, Pingyuan Wang, Paula V. Villarreal, Olivia D. Solomon, Sonja J. Stutz, Aditi, Jacob Porter, Komi Gbedande, Brendan Prideaux, Thomas A. Green, Erin H. Seeley, Parimal Samir, Kelley T. Dineley, Gracie Vargas, Jia Zhou, Irma Cisneros, Robin Stephens, and Fernanda Laezza

**Figure S1**

**
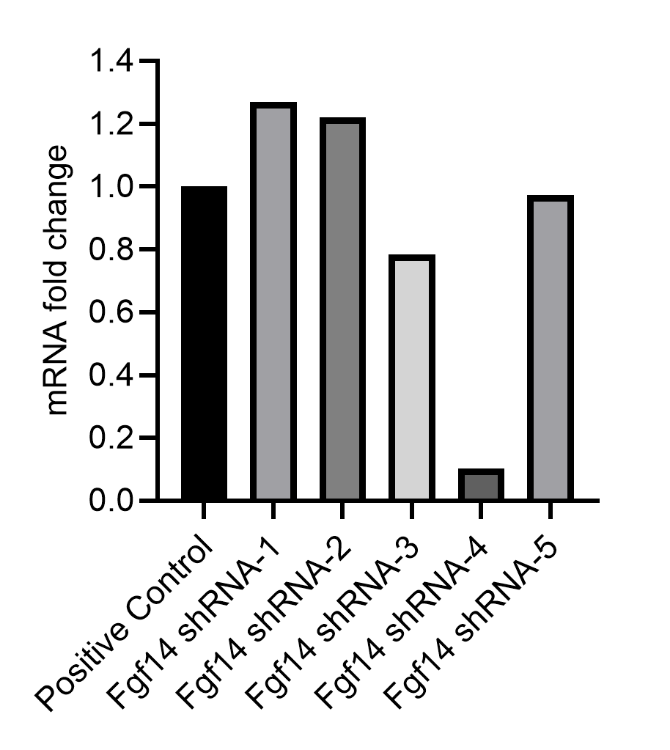
**

**Figure S1: *In vitro* validation of AAV-shFGF14.** qPCR quantification of Fgf14 mRNA in HEK293 cells co-transfected with a plasmid overexpressing FGF14 and shCTRL (positive control) or HEK293 cells co-transfected with a plasmid overexpressing FGF14 and one of five short hairpins targeting the Fgf14 coding region.

**Figure S2**

**
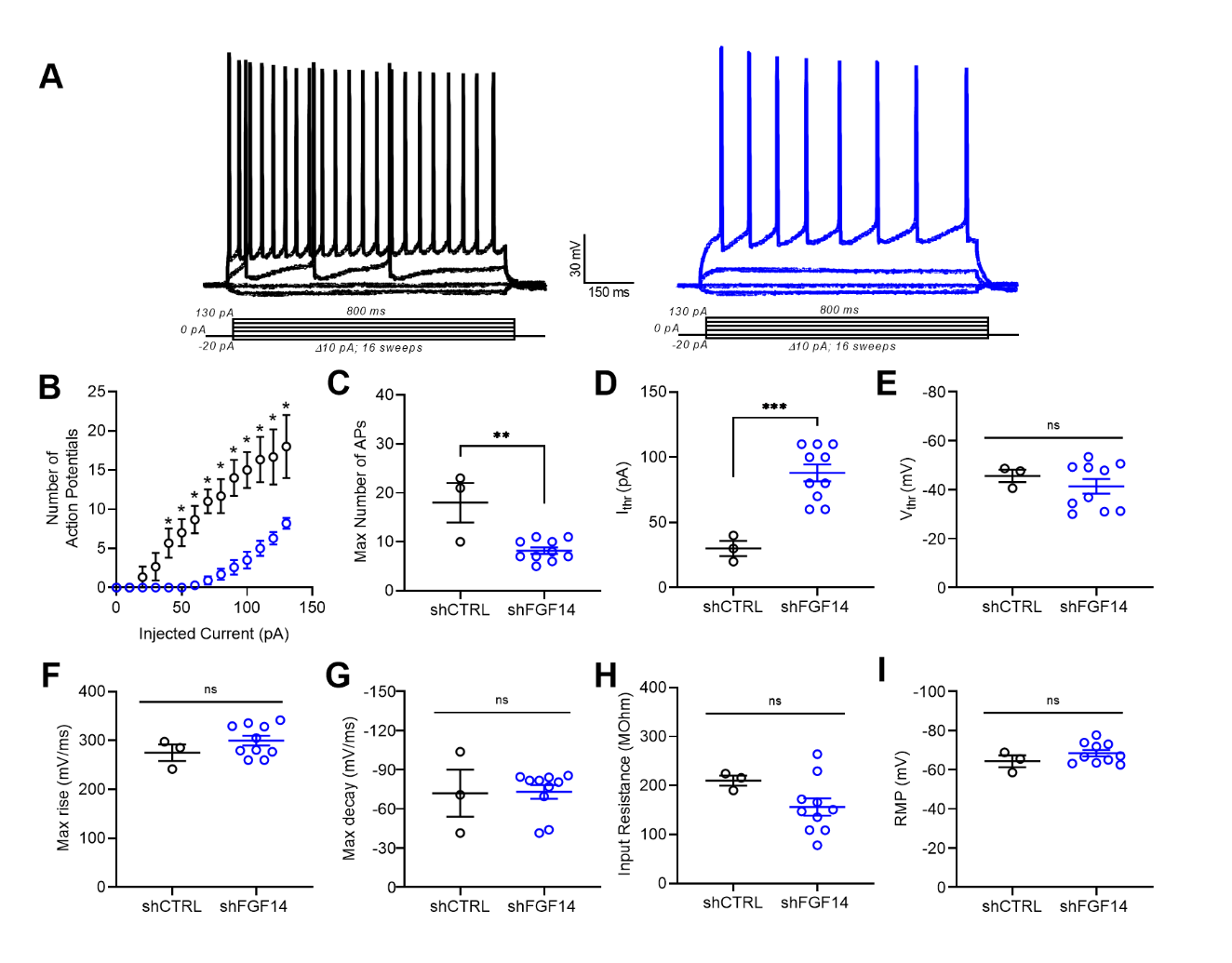
**

**Figure S2: *Ex vivo* validation of the AAV-shFGF14 vector.** (**A**) Representative traces of action potentials fired by CA1 neurons expressing the AAV-shCTRL (black) or AAV-shFGF14 (blue) construct in response to the depicted current clamp protocol.(**B**) Number of action potentials fired by CA1 neurons belonging to the experimental groups described in (**A**) over a range of injected current stimuli. (**C**) Comparison of the maximum number of action potentials fired by CA1 neurons belonging to the indicated experimental groups. (**D**,**E**) Comparison of the current threshold (I_thr_) (**D**) and voltage threshold (V_thr_) (**E**) for the action potential initiation of CA1 neurons belonging to the indicated experimental groups. (**F**,**G**) Comparison of the maximum rise (**F**) and maximum decay (**G**) of action potentials fired by CA1 neurons belonging to the indicated experimental groups. (**H**,**I**) Comparison of the input resistance and resting membrane potential (RMP) of CA1 neurons belonging to the indicated experimental groups. Data are mean ± SEM (n = 3-10 cells/group; slices from N = 2-3 mice per group). Significance was assessed using a Student’s t-test. In (**C**-**I**): ns, not significant; **, p < 0.01; ***, p < 0.001. In (**B**), * denotes current steps at which the number of action potentials fired by CA1 neurons expressing AAV-shFGF14 is significantly lower (p is at least < 0.05) than CA1 neurons expressing AAV-shCTRL.

**Figure S3**

**
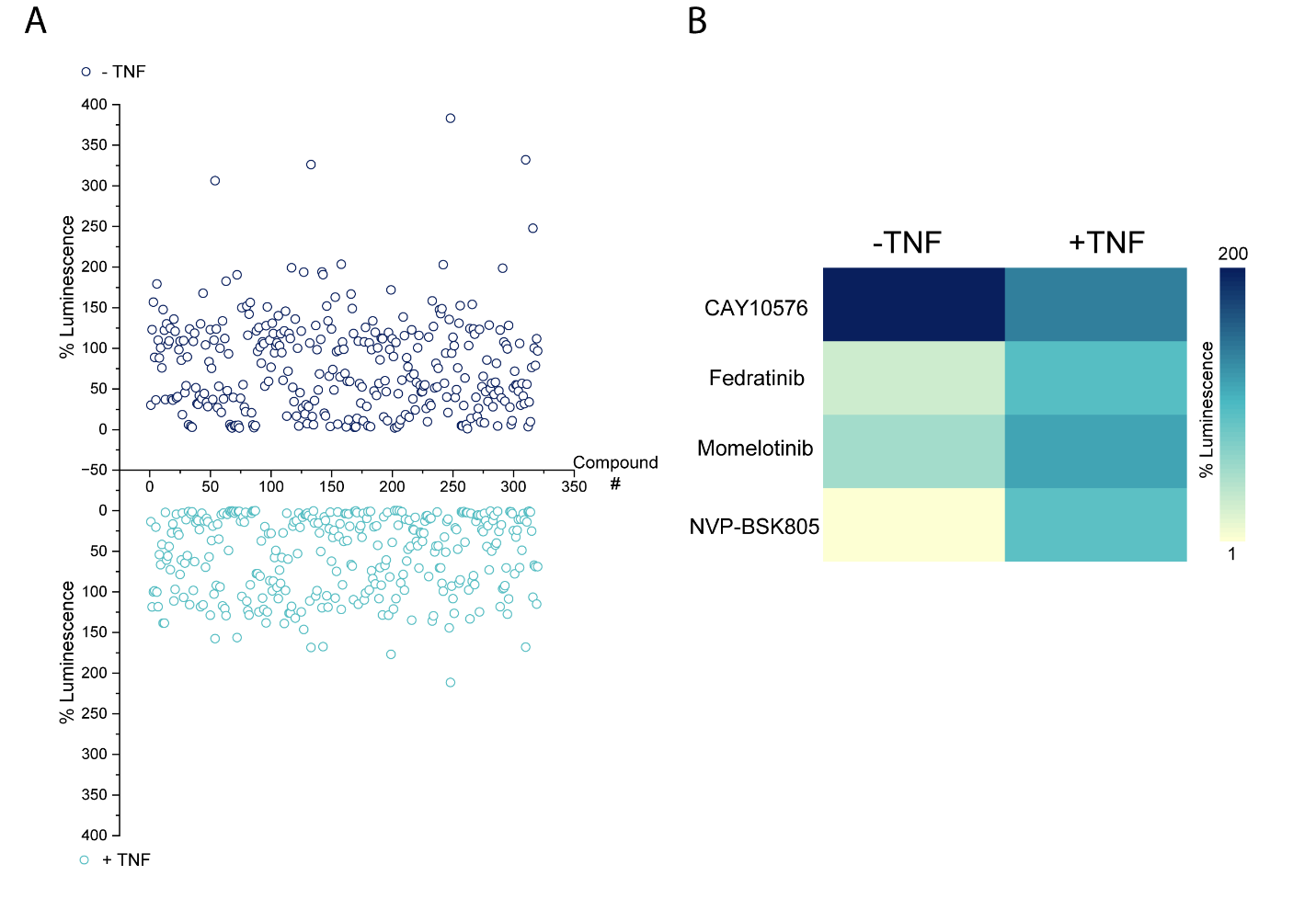
**

**Figure S3: Summary of high throughput screening of Broad Institute Collection against the FGF14:Na_v_1.6 complex with and without TNF stimulation.** (**A**) Effects of 320 compounds from the Broad Institute Collection on FGF14:Na_v_1.6 complex assembly with and without TNF treatment. The compound assigned to each number is listed in Table S5. (**B**) Comparison of three JAK2 inhibitors (Fedratinib, Momelotinib, and NVP-BSK805) and one IKK inhibitor (CAY10576) on FGF14:Na_v_1.6 complex assembly with and without TNF stimulation.

**Figure S4**

**
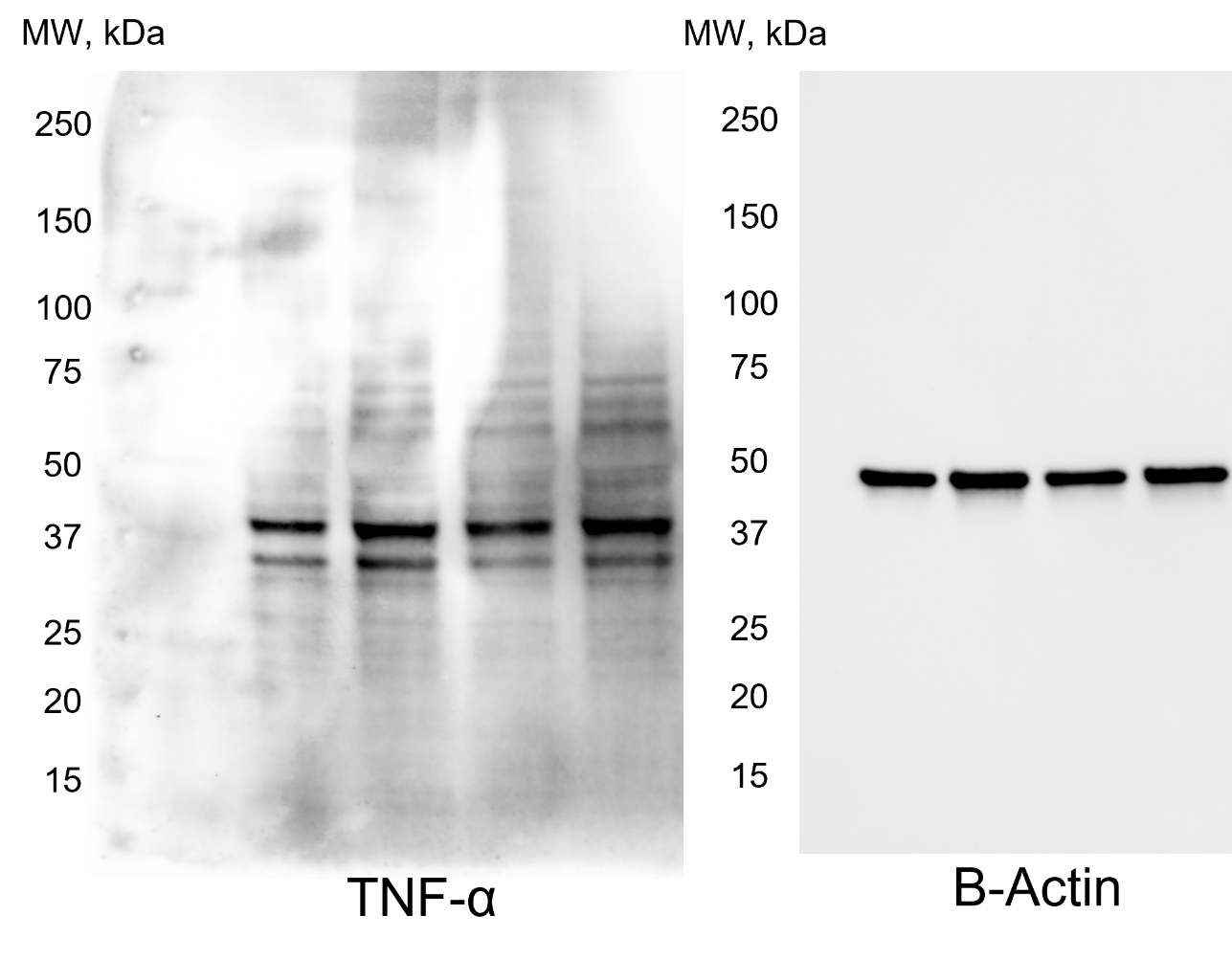
**

**Figure S4: Presence of TNF in hippocampi of *IL*-*10^-/-^* mice infected with *P. chabaudi* on day 7 post-infection.** Left: full, uncropped immunoblot of TNF from hippocampal lysate. Right: full, uncropped immunoblot of B-actin from hippocampal lysate.

**Table S1**

| Condition | Peak *I*_Na_ Density (pA/pF) | V_1/2_ of Activation (mV) | V_1/2_ of Inactivation (mV) |
| --- | --- | --- | --- |
| *Uninfected + vehicle* | -62.9 ± 10.4 (6) | -31.4 ± 1.1 (6) | -61.3 ± 1.8 (6) |
| *Infected + vehicle* | -109.1 ± 11.1 (8)^a^ | -30.4 ± 1.1 (8) | -57.1 ± 2.1 (7) |
| *Infected + 10 µM R-7050* | -53.6 ± 4.9 (11)^b^ | -31.1 ± 0.9 (11) | -57.8 ± 1.5 (7) |
| *Infected + 20 µM Fedratinib* | -58.2 ± 8.1 (6)^c^ | -34.4 ± 1.0 (6) | -60.7 ± 2.8 (6) |
| *Infected + 50 µM ZL181* | -55.0 ± 7.2 (6)^d^ | -33.1 ± 1.4 (6) | -59.9 ± 2.7 (6) |

**Table S1: *P*. *chabaudi* infection increases Na_v_1.6-mediated *I*_Na_ through TNFR1-JAK2 signaling.** Table summary of voltage-clamp data shown in Figure 2. Data are mean ± SEM (n).

^a^ p = 0.0052; Uninfected + vehicle versus Infected + vehicle; one-way ANOVA with post hoc Tukey’s multiple comparisons test.

^b^ p < 0.0001; Infected + vehicle versus Infected + R-7050; one-way ANOVA with post hoc Tukey’s multiple comparisons test.

^c^ p = 0.0018; Infected + vehicle versus Infected + Fedratinib; one-way ANOVA with post hoc Tukey’s multiple comparisons test.

^d^ p = 0.0009; Infected + vehicle versus Infected + ZL181; one-way ANOVA with post hoc Tukey’s multiple comparisons test.

**Table S2**

| Condition | Max Number of Action Potentials | I_thr_ (pA) | V_thr_ (mV) | Max Rise (mV/ms) | Max Decay (mV/ms) |
| --- | --- | --- | --- | --- | --- |
| *Uninfected + vehicle* | 18.5 ± 2.4 (6) | 61.7 ± 15.8 (6) | -36.8 ± 1.6 (6) | 257.4 ± 24.7 (6) | -71.6 ± 6.2 (6) |
| *Infected + vehicle* | 30.3 ± 2.1 (6)^a^ | 41.7 ± 9.1 (6) | -43.1 ± 2.1 (6) | 311.5 ± 21.4 (6) | -80.0 ± 7.4 (6) |
| *Infected + 10 µM R-7050* | 16.7 ± 2.1 (11)^b^ | 74.5 ± 10.4 (11) | -40.9 ± 2.0 (11) | 292.8 ± 13.4 (11) | -66.0 ± 3.3 (11) |
| *Infected + 20 µM Fedratinib* | 14.9 ± 2.0 (11)^c^ | 59.1 ± 6.8 (11) | -38.5 ± 1.6 (11) | 260.5 ± 17.3 (11) | -64.7 ± 6.6 (11) |
| *Infected + 50 µM ZL181* | 14.0 ± 2.1 (10)^d^ | 50.0 ± 8.0 (10) | -39.0 ± 1.3 (10) | 276.5 ± 21.3 (10) | -75.4 ± 3.9 (10) |

**Table S2: *P*. *chabudi* infection increases CA1 pyramidal neuron excitability through TNFR1-JAK2 signaling.** Table summary of current-clamp data shown in Figure 2. Data are mean ± SEM (n).

^a^ p = 0.022; Uninfected + vehicle versus Infected + vehicle; one-way ANOVA with post hoc Tukey’s multiple comparisons tests

^b^ p = 0.0015; Infected + vehicle versus Infected + R-7070; one-way ANOVA with post hoc Tukey’s multiple comparisons test.

^c^ p = 0.0003; Infected + vehicle versus Infected + Fedratinib; one-way ANOVA with post hoc Tukey’s multiple comparisons test.

^d^ p = 0.0002; Infected + vehicle versus Infected + ZL181; one-way ANOVA with post hoc Tukey’s multiple comparisons test.

**Table S3**

| Condition | Max Number of Action Potentials | I_thr_ (pA) | V_thr_ (mV) | Max Rise (mV/ms) | Max Decay (mV/ms) |
| --- | --- | --- | --- | --- | --- |
| *Uninfected (no treatment)* | 16.7 ± 1.0 (9) | 57.8 ± 10.5 (9) | -50.8 ± 1.3 (9) | 305.9 ± 28.9 (9) | -67.8 ± 7.5 (9) |
| *Infected (no treatment)* | 26.6 ± 2.0 (7)^a^ | 48.6 ± 8.8 (7) | -50.6 ± 2.7 (7) | 287.2 ± 14.4 (7) | -68.7 ± 4.5 (7) |
| *Infected + anti-IgG* | 25.7 ± 2.0 (7)^b^ | 38.6 ± 4.0 (7) | -44.9 ± 1.7 (7) | 342.2 ± 20.1 (7) | -87.7 ± 2.8 (7) |
| *Infected + anti-TNF* | 16.3 ± 1.8 (7)^c^ | 72.9 ± 9.9 (7) | -48.4 ± 1.1 (7) | 348.3 ± 18.4 (7) | -65.2 ± 6.5 (7) |

**Table S3. *In vivo* neutralization of TNF mitigates the hyperexcitability phenotype induced by *P*. *chabaudi* infection.** Table summary of current-clamp data shown in Figure 3. Data are mean ± SEM (n).

^a^ p = 0.0012; Uninfected (no treatment) versus Infected (no treatment); one-way ANOVA with post hoc Tukey’s multiple comparisons test.

^b^ p = 0.0031; Uninfected (no treatment) versus Infected + anti-IgG; one-way ANOVA with post hoc Tukey’s multiple comparisons test.

^c^ p = 0.0015; Infected (no treatment) versus Infected + anti-TNF; one-way ANOVA with post hoc Tukey’s multiple comparisons test.

**Table S4**

| Condition | Max Number of Action Potentials | I_thr_ (pA) | V_thr_ (mV) | Max Rise (mV/ms) | Max Decay (mV/ms) |
| --- | --- | --- | --- | --- | --- |
| *Uninfected + shCTRL* | 20.3 ± 1.4 (6) | 35.0 ± 2.2 (6) | -41.0 ± 2.4 (6) | 295.2 ± 16.4 (6) | -84.0 ± 3.9 |
| *Infected + shCTRL* | 31.0 ± 2.5^a^ (6) | 25.0 ± 3.4 (6) | -43.6 ± 2.9 (6) | 295.6 ± 27.8 (6) | -68.6 ± 12.8 (6) |
| *Infected + shFGF14* | 18.7 ± 2.5^b^ (6) | 36.7 ± 4.2 (6) | -46.2 ± 3.3 (6) | 327.0 ± 18.2 (6) | -76.1 ± 3.4 (6) |

**Table S4. *In vivo* genetic silencing of FGF14 is sufficient to block hyperexcitability of CA1 pyramidal neurons caused by *P*. *chabaudi* infection.** Table summary of current-clamp data show in Figure 4. Data are mean ± SEM (n).

^a^ p 0.0091; Uninfected + shCTRL versus Infected + shCTRL; one-way ANOVA with post hoc Tukey’s multiple comparisons test.

^b^ p = 0.0031; Infected + shCTRL versus Infected + shFGF14; one-way ANOVA with post hoc Tukey’s multiple comparisons test.

**Figure S5**


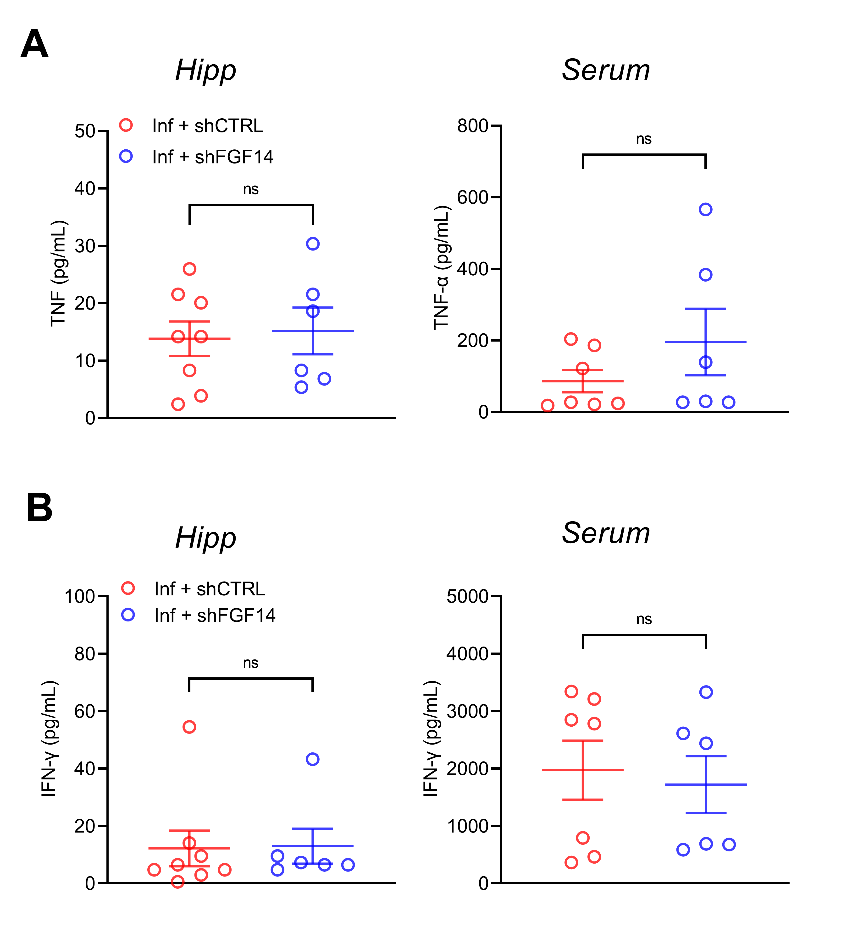


**Figure S5: Genetic silencing of FGF14 in CA1 neurons does not affect TNF or IFN-γ production.** (**A**) Comparison of protein levels of TNF between the indicated experimental groups in the hippocampus and serum. (**B**) Comparison of protein levels of IFN-γ between the indicated experimental groups in the hippocampus and serum. Data are mean ± SEM (n = 6-8 replicates per group). Statistical significance was assessed using a Student’s t-test: ns, not significant.

**Table S5**

| 1 | 968 |
| --- | --- |
| 2 | (+)­Etomoxir(sodiumsalt) |
| 3 | (+/-)-Blebbistatin |
| 4 | 16-beta-bromoandrosterone |
| 5 | 17-AAG |
| 6 | A 804598 |
| 7 | abiraterone |
| 8 | ABT-199 |
| 9 | ABT-263 (Navitoclax) |
| 10 | ABT-737 |
| 11 | AC 55649 |
| 12 | AC 55649 |
| 13 | afatinib |
| 14 | AGK 2 |
| 15 | alisertib |
| 16 | AM 580 |
| 17 | AM-580 |
| 18 | Apicidin |
| 19 | AT13387 |
| 20 | AT-406 |
| 21 | AT406 (SM- 406, ARRY- 334543) |
| 22 | AT7867 |
| 23 | axitinib |
| 24 | AZ-3146 |
| 25 | azacitidine |
| 26 | AZD1480 |
| 27 | AZD4547 |
| 28 | AZD6482 |
| 29 | AZD7545 |
| 30 | AZD7762 |
| 31 | AZD8055 |
| 32 | bafilomycin A1 |
| 33 | barasertib |
| 34 | bardoxolone methyl |
| 35 | Bax channel blocker |
| 36 | BEC |
| 37 | belinostat |
| 38 | bexarotene |
| 39 | BI-2536 |
| 40 | BIBR-1532 |
| 41 | BIRB-796 |
| 42 | birinapant |
| 43 | BIX01294(hydrochloride hydrate) |
| 44 | bleomycin A2 |
| 45 | BMS 195614 |
| 46 | BMS-270394 |
| 47 | BMS-536924 |
| 48 | BMS-754807 |
| 49 | bortezomib |
| 50 | bosutinib |
| 51 | BRD 9876 |
| 52 | BRD1812 |
| 53 | BRD4770 |
| 54 | BRD-K70511574 (HMN-214) |
| 55 | brefeldin A |
| 56 | brivanib |
| 57 | BYL-719 |
| 58 | C6-ceramide |
| 59 | cabozantinib |
| 60 | CAL-101 |
| 61 | canertinib |
| 62 | carboplatin |
| 63 | CAY10576 |
| 64 | CAY10594 |
| 65 | CAY10603 |
| 66 | CCT036477 |
| 67 | CD 1530 |
| 68 | CD-437 |
| 69 | cediranib |
| 70 | Ceranib­2 |
| 71 | ceranib-2 |
| 72 | Cerulenin |
| 73 | Ch 55 |
| 74 | Ch 55 |
| 75 | CHIR-99021 |
| 76 | CHM 1 |
| 77 | CI 976 |
| 78 | CI-976 |
| 79 | CID 2858522 |
| 80 | CID 5951923 |
| 81 | Cimetidine |
| 82 | Cisplatin |
| 83 | clofarabine |
| 84 | crizotinib |
| 85 | cucurbitacin I |
| 86 | Cucurbitacin I |
| 87 | Curcumin |
| 88 | Cyclosporin A |
| 89 | Cyclosporin A |
| 90 | Cytarabine |
| 91 | cytochalasin B |
| 92 | dabrafenib |
| 93 | dacarbazine |
| 94 | daporinad |
| 95 | dasatinib |
| 96 | decitabine |
| 97 | dexamethasone |
| 98 | dinaciclib |
| 99 | docetaxel |
| 100 | Doxorubicin |
| 101 | epigallocatechin-3-monogallate |
| 102 | erastin |
| 103 | erismodegib |
| 104 | erlotinib |
| 105 | etomoxir |
| 106 | etoposide |
| 107 | EX 527 (Selisistat) |
| 108 | EX-527 |
| 109 | FGIN-1-27 |
| 110 | Flavopiridol (Alvocidib) |
| 111 | fluorouracil |
| 112 | Fluvastatin sodium |
| 113 | foretinib |
| 114 | fulvestrant |
| 115 | fumonisin B1 |
| 116 | Fumonisin B1 |
| 117 | GDC-0879 |
| 118 | GDC-0941 |
| 119 | gefitinib |
| 120 | gemcitabine |
| 121 | GMX-1778 |
| 122 | GSK 4112 |
| 123 | GSK J4 |
| 124 | GSK1059615 |
| 125 | GSK2636771 |
| 126 | GSK461364 |
| 127 | GSK525762A |
| 128 | GW 405833 |
| 129 | GW-405833 |
| 130 | HBX 41108 |
| 131 | HC 067047 |
| 132 | HLI 373 |
| 133 | HMN-214 |
| 134 | Homoharringtonine |
| 135 | hyperforin |
| 136 | ibrutinib |
| 137 | IC-87114 |
| 138 | ifosfamide |
| 139 | imatinib |
| 140 | isoliquiritigenin |
| 141 | ISOX |
| 142 | istradefylline |
| 143 | ISX 9 |
| 144 | JNJ 26854165 |
| 145 | JW 480 |
| 146 | JW 55 |
| 147 | JW-55 |
| 148 | JW-74 |
| 149 | KHS101 |
| 150 | Ki8751 |
| 151 | Ko 143 |
| 152 | Ko-143 |
| 153 | KU 0060648 |
| 154 | KU-0063794 |
| 155 | KU-55933 |
| 156 | KU-60019 |
| 157 | KW-2449 |
| 158 | KX2-391 |
| 159 | L-685,458 |
| 160 | lapatinib |
| 161 | LE 135 |
| 162 | LE 135 |
| 163 | lenvatinib |
| 164 | linifanib |
| 165 | Lovastatin |
| 166 | LRRK2-IN-1 |
| 167 | LY 2183240 |
| 168 | LY-2157299 |
| 169 | manumycin A |
| 170 | Maritoclax |
| 171 | masitinib |
| 172 | methotrexate |
| 173 | MG-132 |
| 174 | MGCD-265 |
| 175 | MI-2 |
| 176 | MIRA-1 |
| 177 | mitomycin |
| 178 | MK-0752 |
| 179 | MK-2206 2HCl |
| 180 | ML029 |
| 181 | ML031 |
| 182 | ML162 |
| 183 | ML203 |
| 184 | ML210 |
| 185 | MLN 2480 |
| 186 | MLN2238 |
| 187 | MLN2480 |
| 188 | MLN4924 |
| 189 | momelotinib |
| 190 | MST-312 |
| 191 | myricetin |
| 192 | myriocin |
| 193 | narciclasine |
| 194 | Necrostatin­7 |
| 195 | necrostatin-7 |
| 196 | necrosulfonamide |
| 197 | nelarabine |
| 198 | neratinib |
| 199 | neuronal differentiation inducer III |
| 200 | nilotinib |
| 201 | nintedanib |
| 202 | NSC 95397 |
| 203 | NSC23766 (hydrochloride) |
| 204 | NSC95397 |
| 205 | nutlin-3 |
| 206 | NVP-ADW742 |
| 207 | NVP-BSK805 |
| 208 | NVP-TAE684 |
| 209 | obatoclax |
| 210 | olaparib |
| 211 | oligomycin A |
| 212 | OSI-027 |
| 213 | OSI-906 (Linsitinib) |
| 214 | OSI-930 |
| 215 | Ouabain |
| 216 | oxaliplatin |
| 217 | pazopanib |
| 218 | PD 153035 |
| 219 | PD318088 |
| 220 | PDMP |
| 221 | pevonedistat |
| 222 | PF 4800567 hydrochloride |
| 223 | PF 750 |
| 224 | PF-3758309 |
| 225 | PF-750 |
| 226 | PHA-793887 |
| 227 | Pifithrin-&alpha; hydrobromide |
| 228 | pifithrin-mu |
| 229 | Pifithrin­α |
| 230 | PIK-93 |
| 231 | Pluripotin |
| 232 | PLX-4720 |
| 233 | PRIMA-1 |
| 234 | PRIMA­1MET |
| 235 | PRL­3 Inhibitor |
| 236 | PRL-3 inhibitor I |
| 237 | Prochlorperazine dimaleate |
| 238 | purmorphamine |
| 239 | PX-12 |
| 240 | PX-12 |
| 241 | PYR 41 |
| 242 | QS 11 |
| 243 | quizartinib |
| 244 | R428 |
| 245 | RAF265 |
| 246 | regorafenib |
| 247 | RG-108 |
| 248 | Rigosertib |
| 249 | RO4929097 |
| 250 | ruxolitinib |
| 251 | S3I-201 (NSC 74859) |
| 252 | salermide |
| 253 | saracatinib |
| 254 | SB 225002 |
| 255 | SB-431542 |
| 256 | SB-525334 |
| 257 | SB-743921 |
| 258 | SB743921 |
| 259 | SCH 529074 |
| 260 | SCH 530348 |
| 261 | SCH 79797 dihydrochloride |
| 262 | SCH 79797 dihydrochloride |
| 263 | Selumetinib (AZD6244) |
| 264 | semagacestat |
| 265 | serdemetan |
| 266 | SGX-523 |
| 267 | SID 2668150 |
| 268 | sildenafil |
| 269 | silmitasertib |
| 270 | Simvastatin |
| 271 | sirolimus |
| 272 | sitagliptin |
| 273 | SKI-II |
| 274 | SN 38 |
| 275 | SNS-032 |
| 276 | sorafenib |
| 277 | sotrastaurin |
| 278 | SP600125 (pyrazolanthrone) |
| 279 | Spautin-1 |
| 280 | SR 1001 |
| 281 | SRT-1720 |
| 282 | STF 31 |
| 283 | SU11274 |
| 284 | sunitinib |
| 285 | tacrolimus |
| 286 | tamatinib (R406) |
| 287 | Tamoxifen |
| 288 | tandutinib |
| 289 | temozolomide |
| 290 | temsirolimus |
| 291 | teniposide |
| 292 | TG-100-115 |
| 293 | TG-101348 |
| 294 | TGX-221 |
| 295 | thalidomide |
| 296 | Tigecycline |
| 297 | Tipifarnib (S enantiomer) |
| 298 | tipifarnib-P1 |
| 299 | tivozanib |
| 300 | topotecan |
| 301 | trametinib |
| 302 | triptolide |
| 303 | TW-37 |
| 304 | UNC 0638 |
| 305 | valdecoxib |
| 306 | vandetanib |
| 307 | veliparib |
| 308 | vemurafenib |
| 309 | VER 155008 |
| 310 | Vincristine |
| 311 | VX-680 |
| 312 | WP1130 |
| 313 | WZ4002 |
| 314 | WZ8040 |
| 315 | XL765 |
| 316 | YK 4-279 |
| 317 | YM-155 |
| 318 | YM155 (Sepantronium Bromide) |
| 319 | Zebularine |
| 320 | ZSTK474 |

**Table S5: Compound ID for data shown in Figure S1.** List identifying the compound name that corresponds to the compound numbers shown in Figure S1.
